# Glycosylation of Quercetin by Selected Entomopathogenic Filamentous Fungi and Prediction of Its Products’ Bioactivity

**DOI:** 10.3390/ijms241411857

**Published:** 2023-07-24

**Authors:** Tomasz Tronina, Mateusz Łużny, Monika Dymarska, Monika Urbaniak, Ewa Kozłowska, Michał Piegza, Łukasz Stępień, Tomasz Janeczko

**Affiliations:** 1 Department of Food Chemistry and Biocatalysis, Wrocław University of Environmental and Life Sciences, Norwida 25, 50-375 Wrocław, Poland; mat.luzny@gmail.com (M.Ł.); monika.dymarska@gmail.com (M.D.); e.a.kozlowska@gmail.com (E.K.); 2 Department of Pathogen Genetics and Plant Resistance, Institute of Plant Genetics, Polish Academy of Sciences, Strzeszyńska 34, 60-479 Poznań, Poland; murb@igr.poznan.pl (M.U.); lste@igr.poznan.pl (Ł.S.); 3 Department of Biotechnology and Food Microbiology, Wrocław University of Environmental and Life Sciences, Chełmońskiego 37, 51-630 Wrocław, Poland; michal.piegza@upwr.edu.pl

**Keywords:** quercetin, biotransformation, entomopathogenic fungi, 4-*O*-methyloglycosylation

## Abstract

Quercetin is the most abundant flavonoid in food products, including berries, apples, cauliflower, tea, cabbage, nuts, onions, red wine and fruit juices. It exhibits various biological activities and is used for medical applications, such as treating allergic, inflammatory and metabolic disorders, ophthalmic and cardiovascular diseases, and arthritis. However, its low water solubility may limit quercetin’s therapeutic potential. One method of increasing the solubility of active compounds is their coupling to polar molecules, such as sugars. The attachment of a glucose unit impacts the stability and solubility of flavonoids and often determines their bioavailability and bioactivity. Entomopathogenic fungi are biocatalysts well known for their ability to attach glucose and its 4-*O*-methyl derivative to bioactive compounds, including flavonoids. We investigated the ability of cultures of entomopathogenic fungi belonging to *Beauveria*, *Isaria*, *Metapochonia*, *Lecanicillium* and *Metarhizium* genera to biotransform quercetin. Three major glycosylation products were detected: (**1**), 7-*O*-β-D-(4″-*O*-methylglucopyranosyl)-quercetin, (**2**) 3-*O*-β-D-(4″-*O*-methylglucopyranosyl)-quercetin and (**3**) 3-*O*-β-D-(glucopyranosyl)-quercetin. The results show evident variability of the biotransformation process, both between strains of the tested biocatalysts from different species and between strains of the same species. Pharmacokinetic and pharmacodynamic properties of the obtained compounds were predicted with the use of cheminformatics tools. The study showed that the obtained compounds may have applications as effective modulators of intestinal flora and may be stronger hepato-, cardio- and vasoprotectants and free radical scavengers than quercetin.

## 1. Introduction

Quercetin, a widely distributed representative of a large class of flavonoid compounds—flavonols—contains five hydroxyl groups at positions 3, 3′, 5, 7 and 4′ of the basic flavone skeleton [1]. Quercetin is commonly found in the plant kingdom and has been identified in many fruits and vegetables [2,3]. For this reason, humans’ daily intake of this flavonoid is relatively high and reaches several milligrams. Apples, blueberries, cranberries and onions are particularly rich sources of quercetin [3,4,5]. In many studies on cell lines and animal models, it has been proven that this flavonol is highly biologically active and has health-promoting activity, e.g., anticancer, antibacterial, antiviral, anti-obesity, antidiabetic, anti-inflammatory, as well as neuroprotective and hepatoprotective. Human studies show that quercetin may play a significant role in preventing coronary artery disease, asthma and Alzheimer’s disease. Quercetin can potentially alleviate mood disorders and improve overall health by boosting the immune system [6,7,8,9,10,11].

Glycosylation of flavonoids is a known method to improve their stability and water solubility. Like their aglycone, quercetin glycosides show multiple biological activities, e.g., neuroprotective, cardioprotective, antioxidative, chemopreventive and antiallergic [12]. A glucoside of quercetin—isoquercetin (3′,4′,5,7-tetrahydroxyflavone 3-*O*-β-D-glucopyranoside)—which occurs in vegetables, herbs and flowers, can potentially be used in the prevention and treatment of numerous disorders and diseases due to its antiviral, antidiabetic and antioxidant properties [13,14]. In the case of the anti-obesity effect, it was found that isoquercetin shows a higher therapeutic effect than its aglycone—quercetin [15]. It is already well established that in the same conditions (temperature and pH value below 7), flavonoid glycosides are more soluble in water than their aglycones. The solubility of glycoside naringin at 20 °C is more than 110-fold higher than its aglycone naringenin [16,17]. The solubility of rutin in water is 125 mg/L [18], whereas, for quercetin aglycone, it is only 0.512 mg/L (more than 240-fold lower) [19]. The presence of a sugar moiety in the flavonoid molecule was proposed to be the crucial determinant of its absorption in humans [20]; however, this strongly depends on the type of conjugated sugar moiety. A study performed on Beagle dogs showed that the bioavailability of 3-*O*-glycoside of quercetin (isoquercetin) was higher (235%), whereas the bioavailability of 3-*O*-glucorhamnoside of quercetin (rutin) was lower (92%) in comparison to its aglycone quercetin [15,21]. In contrast, a study in rats found that isoquercetin bioavailability was 185% that of quercetin, while quercetin 3-rhamnoside was barely absorbed (less than 25%) [22]. The high biological activity and increased bioavailability of isoquercetin encouraged us to obtain glucose and 4-*O*-methyl glucose derivatives of quercetin through whole-cell biotransformation.

Glycosylation is a prominent strategy utilized by organisms from diverse environments to regulate the physicochemical and biological properties of both macro- and micromolecules [23,24,25]. It has been suggested that each organism dedicates up to 1% of its genome encoding proteins to glycosylation processes. Glycosylation inside living cells is mediated mainly by a group of enzymes named glycosyltransferases (GTs) [26,27]. So far, GTs from bacteria, plants and animals have been extensively studied. Despite the great potential of fungi as whole-cell biocatalysts for the glycosylation of various substances [28,29,30,31,32,33,34], only a few fungal GTs, such as MhGT1 from *Mucor hiemalis*, UGT58A1 from *Rhizopus japonicus*, UGT59A1 from *Absidia coerulea* and BbGT86 from *Beauveria bassiana*, have been isolated and characterized [35,36,37].

The main product of quercetin biotransformation conducted in plant cultures was its 3-*O*-glucopyranoside [38,39]. Isoquercetin was also the major product in the fungal strain *Cunninghamella elegans* ATCC 9245 [40]. As a result of quercetin biotransformation by the strain of *Gliocladium deliquescens* NRRL 1086, along with the predicted product quercetin 3-*O*-β-D-glucoside (isoquercetin), three additional metabolites—2-protocatechuoyl-phlorogucinol carboxylic acid, 2,4,6-trihydroxybenzoic acid and protocatechuic acid—were also isolated. The time-course experiments revealed two metabolic routes—regioselectivity glycosylation and quercetin 2,3-dioxygenation—co-existing in this culture [41]. A similar pathway of quercetin biotransformation was observed in the culture of a *Bacillus cereus* strain [42]. There are also other bacterial strains capable of degrading quercetin (*Rhizobium loti* and *Bradyrhizobium* strains (*Lotus*)) [43]. The biotransformation of quercetin by *Streptomyces griseus* (ATCC 13273) resulted in the isolation and characterization of five hydroxylated and/or methylated metabolites [44].

Quercetin-4″-*O*-methyl-7-*O*-β-D-glucopyranoside was the main product isolated after the biotransformation of quercetin in *Beauveria bassiana* ATCC 7159 [45]. The ability of strains of this species to regioselectively (C-7) attach 4-*O*-methylglucopyranose was also confirmed in later studies [5]. The attachment of methylated glucose was also observed in the culture of the *Isaria fumosorosea* KCH J2 strain, but at the C-3 position, which resulted in obtaining 4″-*O*-methyl-isoquercetin [2]. Employing genome mining, Xie et al. found that this GT-MT module is not exclusive to *B. bassiana* but is also present in other Hypocreales fungi, such as *Isaria fumosorosea*, *Claviceps purpurea*, *Cordyceps militaris* and *Metarhizium robertsii* [24]. Interestingly, the same glucosyltransferase from a particular entomopathogen is able to catalyze different glycosylation reactions. The glycosyltransferase gene (BbGT) from *B. bassiana* ATCC 7159 was expressed in other microbial hosts, including *Saccharomyces cerevisiae*, *Escherichia coli, Pseudomonas putida* and *Pichia pastoris*, giving different products. The major glycosylation product of quercetin in *E. coli*, *P. putida* and *P. pastoris* was quercetin-7-*O*-β-D-glucoside, while the enzyme mainly produced quercetin-3-*O*-β-D-glucoside in *S. cerevisiae* [46]. All these observations undoubtedly indicate that living organisms, including entomopathogenic filamentous fungi, are still an underexploited repository of novel GTs, which awaits discovery.

Since the main metabolites of flavonoids obtained from entomopathogenic fungi are their glucose derivatives, it was a natural choice to use this group of filamentous fungi as catalysts for the biotransformation of quercetin to obtain its glycosides and 4-*O*-methylglycosides. The strains from *Beauveria bassiana* and *Isaria fumosorosea* species have a unique capacity for 4-*O*-methylglycosylation of flavonoids. We conducted research to assess the biotransformation potential of various strains of entomopathogenic fungi, including those with a confirmed capacity for methylglycosylation of flavonoid compounds, as well as strains isolated from insect carcasses and utilized for biotransformations for the first time.

## 2. Results and Discussion

Based on the previously described results regarding the biotransformations of flavonoid compounds in entomopathogenic strains, especially quercetin biotransformation conducted by those micro-organisms, we decided to use five strains of the *Beauveria bassiana* species, two of the *Beauveria caledonica* species and the *Isaria farinosa* KCh KW1.1 as biocatalysts. Among entomopathogenic filamentous fungi, *B. bassiana* strains are most commonly used in biotransformation (including flavonoid compounds) [47,48,49,50]. Our previous publications described differences in the biotransformation of methoxyflavones and dehydroepiandrosterone (DHEA) in the cultures of various strains belonging to this species [51,52]. In this work, we decided to check whether the biotransformation of quercetin in multiple strains of this species will result in the appearance of different products. As one of the biocatalysts, *Beauveria felina* ENC3 was used. To the best of our knowledge, the culture of this species applied as a biocatalyst of flavonoid compounds has never been reported before. In addition, we decided to check the ability of other entomopathogenic strains to biotransform quercetin: two strains of the *Isaria tenuipes* species and *Lecanicillium lecanii*, as well as *Metapochonia bulbillosa* CYS17 and *Metarhizium anisopliae* MU4 (genetic identification is described in the Materials and Methods section).

**Scheme 1:**
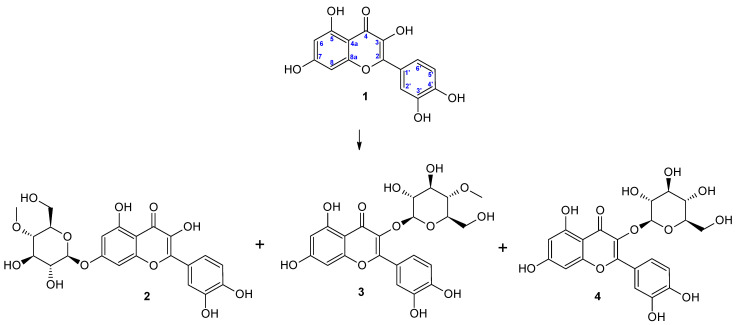
Products (7-*O*-β-D-(4″-O-methylglucopyranosyl)-quercetin (**2**), 3-*O*-β-D-(4″-O-methylglucopyranosyl)-quercetin (**3**), 3-*O*-β-D-(glucopyranosyl)-quercetin (**4**)) obtained during biotransformation of quercetin in cultures of entomopathogenic strains.

The *Isaria fumosorosea* KCH J2 strain has already been described in our previous papers as an effective biocatalyst of flavanones and flavones, including quercetin [2,50,52,53,54]. Based on the results previously described for this strain, we noticed the similarity with *Beauveria bassiana* strains in the transformation of flavonoid compounds. In both cases, the main products were 4-*O*-methyl-glucopyranosides, with the difference that in the case of *B. bassiana*, a hydroxyl group at C-7 was being glycosylated, whereas *I. fumosorosea* cultures attached the sugar moiety to the hydroxyl group at C-3. In addition, the latter species, along with methylglucoside, also produces isoquercetin. On this basis, we used *Isaria fumosorosea* KCh J2 and *B. bassiana* AM278 to obtain reference quercetin metabolites. We obtained one glycoside (**2**) in the culture of the *B. bassiana* AM278 and two glycosides (**3**, **4**) in the *I. fumosorosea* KCh J2 strain culture; the products have been characterized spectroscopically in our earlier publications [2,5] (Scheme 1).

Based on the experiments conducted, we found that the pentahydroxyflavone quercetin used as a substrate is transformed to a variable extent depending on the biocatalyst used. In the cultures of the strains *M. bulbillosa* CYS17, *B. felina* ENC3, *L. lecanii* DSM 63098 and *L. lecanii* NK3, even after ten days of substrate incubation, no glycosylation products were observed (Table 1). Quercetin remained in the reaction medium, proving this polyphenol’s high stability in these experimental conditions.

The products were also not observed in the culture of the *B. caledonica* KCh J3.4 strain. However, in the culture of another strain of the same species, *B. caledonica* KCh J3.3, after seven days of incubation, we identified 3,3′,4′,5-tetrahydroxyflavone 7-*O*-β-D-(4″-*O*-methyl)-glucopyranoside (**2**); a compound also formed as the only product in the culture of the *B. bassiana* AM278 strain [5]. Similar differences in the conversion of this substrate were observed during biotransformation in cultures of other *B. bassiana* strains. In the cultures of the KCh J1.5 and KCh BBT strains, we observed, in accordance with the literature data [51], effective 4-*O*-methylglycosylation leading to the formation of compound **2**. However, in the cultures of *B. bassiana* strains KCh J2.1 and KCh J1, even after 10 days of incubation, more than 90% of the substrate remained unchanged. On the other hand, in the culture of the *B. bassiana* KCh J3.2 strain, after three days of biotransformation, we observed the decomposition of the substrate used and a slight increase in compound **2** in the reaction mixture (Table 1).

The most effective biocatalysts producing 7-*O*-β-D-(4′-O-methyl)-glucopyranoside quercetin (**2**) were the strains *B*. *bassiana* KCh J1.5 and *B*. *bassiana* KCh BBT. On the first day, both cultures showed the same ability to produce glycoside 2, above 30% (*p* value > 0.05; Figure S24—Supplementary Materials); however, from the third day of biotransformation, the most effective strain among all tested mushrooms was *B*. *bassiana* KCh J1. 5 (*p* value < 0.05; Figure S24—Supplementary Materials). All the tested fungi belonging to the *Beauveria* genera showed significant differences in the ability to produce metabolite **2** (*p* value < 0.05), with *B*. *bassiana* J2.1 as the least active strain, which produced less than 3% of glycoside **2** after 10 days (Table S2—Supplementary Materials). The results obtained demonstrate that the biocatalytic capacity to produce metabolite **2** in the culture of the tested fungi of the genus *Beauveria* does not depend only on the species (*B*. *caledonica* KCh J3.3 produced significantly lower amounts of glycoside **2** than *B*. *bassiana* KCh J1.5 and *B*. *bassiana* KCh BBT) (*p* value < 0.05; Figure S24—Supplementary Materials) but also on the strain used; after 10 days, *B*. *bassiana* KCh KW 1.1 produced 35-fold less and 25-fold less glycoside **2** than *B*. *bassiana* KCh J1.5 and *B*. *bassiana* KCh BBT, respectively (Table S2—Supplementary Materials). Fungi of the *Isaria* genus demonstrated similarly low biocatalytic efficiency in producing metabolite **2,** as mentioned earlier for the *B. bassiana* KCh J2.1 strain. No significant differences in obtaining metabolite **2** were observed until the seventh day of biotransformation (*p* value > 0.05; Figure S24—Supplementary Materials).

The two-way course of quercetin biotransformation (regioselective glycosylation and 2,3-dioxygenation of quercetin) was previously described in the literature, mainly in bacterial cultures [41,42,43]. Oxidation leading to three metabolites has previously been reported: 2-protocatechuoyl-phlorogucinol carboxylic acid, 2,4,6-trihydroxybenzoic acid and protocatechuic acid. In the culture of *B. bassiana* KCh J3.2, the glycosylation product was 3,3′,4′,5-tetrahydroxyflavone 7-*O*-β-D-(4″-*O*-methyl)-glucopyranoside (**2**). However, in the culture of the *Metarhizium anisopliae* MU4 strain, despite the partial decomposition of the substrate, we obtained 3′,4′,5,7-tetrahydroxyflavone 3-*O*-β-D-(4″-*O*-methyl)-glucopyranoside (**3**).

In the culture of the *I. farinosa* KCh KW 1.1 strain, three glycosylation products were formed: 7-*O*-β-D-(4″-*O*-methylglucopyranosyl)-quercetin (**2**), 3-*O*-β-D-(4″-*O*-methylglucopyranosyl)-quercetin (**3**), 3-*O*-β-D-(glucopyranosyl)-quercetin (**4**). Decomposition products were also present in the reaction mixture, but the unreacted substrate was the dominant compound, even after ten days. Based on the obtained results, it can be concluded that the cells of this strain produce enzymes analogous to both *B. bassiana*—formation of compound **2**—and *I. fumosorosea* species—compounds **3** and **4** [2]. Additionally, three products (**2**, **3** and **4**) but with much higher conversion were formed in cultures of strains *I. tenuipes* MU35 and *I. tenuipes* CYS30. The products accounted for about 90% of the reaction mixture, and the dominant compound was 3-*O*-β-D-(glucopyranosyl)-quercetin (**4**). Interestingly, strain *I*. *tenuipes* MU35 proved to be the most efficient biocatalyst in the production of glycoside **4** for the first 7 days of biotransformation (*p* value < 0.05; Figure S26—Supplementary Materials); however, by day 10 of biotransformation, there was no longer a significant difference in the production of metabolite **4** between *I. tenuipes* MU35 and *I*. *tenuipes* CYS30 (progress in the production of metabolites **3** and **4** with statistical differences available in Supplementary Materials—Figures S25 and S26).

To evaluate the physicochemical properties, pharmacokinetics and potential biological activity of quercetin glycosides compared to quercetin, cheminformatics tools, such as SwissADME and passOnline, were used. Physicochemical descriptor calculations were performed for the substrate and the three products obtained: **2**, **3** and **4**. The predictions were made for ADME parameters (absorption, distribution, metabolism, excretion), pharmacokinetic properties and suitability for medicinal chemistry. The analysis was performed using the online tool SwissADME (Available online: http://www.swissadme.ch (accessed on 30 May 2023)), developed and managed by the Molecular Modeling Group of the Swiss Institute of Bioinformatics (SIB) [55]. Based on the results obtained using this tool, it was found that all the products have significantly lower lipophilicity and significantly higher water solubility than quercetin (**1**) (Table 2).

According to the results we obtained from in silico (i.e., computational) pharmacokinetic predictions, both the test substrate (**1**) and the obtained products (**2**, **3**, **4**) should not passively penetrate the blood/brain barrier. It was also determined that quercetin (**1**) can penetrate the intestine/blood barrier passively, while the described glycosides cannot passively cross this barrier [56]. The attachment of a sugar unit impacts the stability and solubility of flavonoids and often also determines their bioavailability and bioactivity [57,58,59], increases their hydrophilicity and thus affects their bioavailability [60]. Our chemoinformatic studies showed that the 4’-*O*-methylglucoside- and 3-*O*-glucoside of quercetin have the same bioavailability, which may suggest that the presence of a methyl group in the glucose molecule in quercetin glucosides does not affect bioavailability. However, as presented above (Table 2), in the case of the obtained glucosides, the predicted gastrointestinal absorption is low, unlike in the case of quercetin. The results do not support the generally accepted thesis that quercetin glucosides are much better absorbed than their aglycone. However, studies on the bioavailability of quercetin 3-*O*-glycoside (isoquercetin) have shown a significant improvement in absorption for the glycoside [15,21]. The differences in the results of in silico evaluation and results obtained in vivo are probably related to the methodology of determination of the physicochemical properties concerning only the characteristic fragments of the molecule (descriptors) in in silico studies. As a result, the predicted increased hydrophilicity of quercetin glucosides simultaneously results in decreased lipophilicity of the molecules, which may reduce affinity for biological membranes and impede passive transport across the intestinal barrier. Based on in silico studies, glycosylation may inhibit the passive permeation of glucosides into the bloodstream [55]. In vivo studies show the opposite tendency, namely that glycosylation may significantly improve the bioavailability of quercetin; however, this strongly depends on the type of sugar attached. For isoquercetin (glucoside) and rutin (rutoside), the relative total bioavailability of quercetin (i.e., conjugated quercetin and conjugated methyl ethers of quercetin) was 148% and 23%, respectively, compared with quercetin aglycone [61]. In humans, rats and pigs, the absolute bioavailability (i.e., the fraction of an ingested compound that reaches systemic circulation) of unchanged quercetin was only 5% and 1%, respectively [21,62,63]. Certain glycosides, such as quercetin-3-*O*-glucoside (isoquercetin), are substrates for the small intestinal brush border enzyme lactase-phlorizin hydrolase (EC 3.2.1.108) [64]. Quercetin can be enzymatically released from the glucoside form to its aglycone (quercetin), thus being absorbed mainly in the small intestine. This results in relatively high bioavailability of quercetin from isoquercetin [21].

Only about 3% of the ingested quercetin was excreted in urine as quercetin aglycone or its conjugates, which indicates that quercetin is extensively metabolized in the human liver and other organs and by the colonic microflora [62]. In addition, substantial variation between different individuals in the measured quercetin bioavailability parameters was observed by examining published human intervention studies where the sources of quercetin were food, beverages or supplements. From the studies reported so far, the reasons or causes of the interindividual differences are not clear, but based on the known metabolic pathways, it is predicted that dietary history, genetic polymorphisms and variations in gut microbiota metabolism play significant roles [65].

It is known that quercetin (**1**) can significantly alter the composition of the intestinal flora in rats with hyperlipidemia (HLP), increasing the number of beneficial bacteria and decreasing the composition of harmful bacteria by weakening the *Firmicutes/Bacteroidetes* ratio [66]. Quercetin (**1**) and its metabolites can reduce lipid levels and improve liver function; the potential mechanism may be the regulation of metabolism and intestinal flora [67]. In addition, the described glycosides (**2**, **3**, **4**) should not be inhibitors of monooxygenases, which are necessary for the proper functioning of the human body. Based on the predicted pharmacokinetic and pharmacodynamic data (Table 2), 7-*O*-β-D-(4″-*O*-methylglucopyranosyl)-quercetin (**2**), 3-*O*-β-D-(4″-*O*-methylglucopyranosyl)-quercetin (**3**), and 3-*O*-β-D-(glucopyranosyl)-quercetin (**4**) can be considered as potential drugs [56].

Based on the evaluation using the platform Way2Drug PASS Online (http://www.way2drug.com/PASSOnline/predict.php) (accessed on 30 May 2023), the described glycosides with very high probability should, in many cases, show predicted biological activity higher than quercetin (**1**). Table 3 shows the bioinformatic predictions of the most probable biological activities of the obtained glycosides and quercetin (**1**). These activities are in agreement with studies conducted empirically and show that quercetin (**1**) could act against cardiovascular diseases (CVDs) through various mechanisms [68,69,70,71,72,73,74,75]. It was also confirmed in in vitro and in vivo studies that some effects of quercetin (**1**) were slightly higher than those of quercetin glucoside, while in vitro and ex vivo anticoagulant effects of quercetin (**1**) were weaker than those of quercetin glucoside due to their structural characteristics. Moreover, these effects, along with the lack of impairment of vital hemostasis in mice, suggest that quercetin and quercetin-3-*O*-β-D-glucoside can potentially be therapeutic agents for CVDs [76]. Based on data collected in Table 3, similar activity should also be exhibited by quercetin methylglycosides; however, to confirm these activities, it is crucial to perform in vitro and in vivo studies.

The average daily intake of quercetin is estimated at 25 mg to 500 mg per day [77], and administration of flavonols from the diet, such as quercetin or kaempferol, at concentrations as high as 3000 mg/kg has not shown significant toxicity in animal studies and clinical trials [78,79]. The acute toxicity in rats predicted by GUSAR (https://www.way2drug.com/gusar (accessed on 30 May 2023)) is in agreement with the experimental studies cited above. In our computational studies, LD_50_ from the oral route of administration of quercetin (**1**) was determined to be 1892 mg of compound per kg of rat body weight. For compounds **2**, **3** and **4**, the LD_50_ values were higher: 2759, 2494 and 3425 mg/kg. Through the intraperitoneal route of administration, the LD_50_ values for quercetin (**1**) and its glycosides (**2**, **3**, **4**) were determined to be 920, 239, 234 and 582 mg/kg, respectively. Our predictions indicate low toxicity of quercetin and similar safety of quercetin glycosides (**2**, **3**, **4**).

## 3. Materials and Methods

### 3.1. Biotransformation Procedure

Erlenmeyer flasks (300 mL), each containing 100 mL of the sterile cultivation medium (3% glucose, 1% peptone) (POCH, Gliwice, Poland), were inoculated with a suspension of each entomopathogenic strain and then incubated for 3 days at 24 °C on a rotary shaker. After this time, 10 mg of a substrate (quercetin (**1**) was purchased from Sigma-Aldrich (St. Louis, MO, USA)) dissolved in 1 mL of dimethyl sulfoxide (DMSO) was added to the interior. Samples were collected on the first, third, seventh and tenth day of the process (it was repeated in three independent experiments). Then, all products were extracted using ethyl acetate, and the extracts were dried using anhydrous MgSO_4_, concentrated in vacuo and analyzed using TLC and HPLC methods. Quantitative analyses of the mixtures were performed by means of HPLC. The calibration curves for quantitative analyses were prepared using quercetin and its glucosides as standards.

### 3.2. Product Samples

For the scale-up process, Erlenmeyer flasks (2000 mL) were used, each containing 500 mL of the same cultivation medium (3% glucose, 1% peptone; POCH, Gliwice, Poland), which were inoculated in the same way as described above. Three days after inoculation, 100 mg of a substrate was dissolved in 2 mL of DMSO and added to the interior. Samples were collected on the fourteenth day of the process. The products were extracted three times using ethyl acetate and then analyzed using TLC, HPLC and NMR spectroscopy (^1^H NMR, ^13^C NMR, COSY, HMBC and HSQC) analysis (Figures S3, S4, S7–S10, S13–S16, S19–S22—Supplementary Data).

### 3.3. Analysis

The initial tests were carried out using TLC plates (SiO_2_, DC Alufolien Kieselgel 60 F_254_ (0.2 mm thick), Merck, Darmstadt, Germany). The mobile phase contained a mixture of chloroform and methanol in a 9:1 (*v*/*v*) relation. The plates were observed using a UV lamp (254 and 365 nm). The scale-up biotransformation products were separated using 1000 µm preparative TLC silica gel plates (Anatech, Gehrden, Germany). The mobile phase contained a mixture of chloroform and methanol in a 9:1 (*v*/*v*) ratio. The separation products were scraped out and extracted twice using ethyl acetate.

### 3.4. Micro-Organisms

The micro-organisms *Beauveria bassiana* KCh J1.5, KCh J2.1, KCh J1, KCh J3.2 and KCh BBT, *B. caledonica* KCh J3.3 and KCh J3.4, *Isaria farinosa* KCh KW 1.1 and *I. fumosorosea* KCh J2. were obtained from the collection of the Department of Food Chemistry and Biocatalysts, Wrocław University of Environmental and Life Sciences (Wrocław, Poland). The isolation and identification procedures were described in our previous papers [51,60,80]. The strains *Lecanicillium lecanii* NK3 and *L. lecanii* DSM 63098 were obtained from the collection of the Department of Biotechnology and Food Microbiology, Wrocław University of Environmental and Life Sciences (Wrocław, Poland). Strain ENC3 was characterized in a previous paper by Urbaniak et al. (2020) [81] and was placed in the fungi collection of the Institute of Plant Genetics, Polish Academy of Sciences, Poznań, Poland.

DNA extraction and molecular identification of fungal strains: Strains CYS17, CYS30, ENC3, MU35 and MU4 were isolated from insects found in Poland. A modified method using CTAB (hexadecyltrimethylammonium bromide) was applied for genomic DNA extraction, as described before [82]. Species identification was performed on the basis of the sequence analysis of the Internal Transcribed Spacers of the ribosomal DNA region (ITS1-ITS2). Polymerase chain reactions (PCRs) were performed as described previously [83] using DreamTaq Green DNA polymerase (Thermo Scientific, Espoo, Finland). For the PCR amplification, specific primers were used: ITS4—forward primer (5′-TCCTCCGCTTATTGATATGC-3′) and ITS5—reverse primer (5′-GGAAGTAAAAGTCGTAACAAGG-3′) [84]. The amplicons were separated in 1.5% agarose gel (Invitrogen) with GelGreen Nucleic Acid Stain (Biotium Inc. Freemont, CA, USA). For sequence analysis, PCR-amplified DNA fragments were purified as described before [85]. DNA fragments were labeled using a forward primer and the BigDyeTerminator 3.1 kit (Applied Biosystems, Foster City, CA, USA), according to the producer’s recommendations, and precipitated with 96% ethanol. Sequence reading was performed using Applied Biosystems equipment. Sequences were analyzed using the BLASTn algorithm against the GenBank database-deposited reference sequences (standard databases, highly similar sequences—megablast) (Table 4 and Table S1 in Supplementary Data). After molecular identification, fungal strains were placed in the fungi collection of the Institute of Plant Genetics, Polish Academy of Sciences, Poznań, Poland.

### 3.5. HPLC

HPLC analyses were performed on an Ultimate 3000 UHPLC+ focused instrument (Thermo Scientific, Waltham, MA, USA) with a photodiode array detector (detection from 210 to 450 nm wavelength) using an Agilent Zorbax Eclipse XDB-C18 (4.6 mm × 250 mm, 5 µm, Agilent, Santa Clara, CA, USA) at a flow rate of 1.5 mL/min and the following elution program: gradient elution from 0 to 6 min (80% A → 70% A), from 6 to 8 min (70% A → 60% A); gradient elution from 8 to 13 min (60% A → 5% A); isocratic elution from 13 to 16 min (5% A); gradient elution from 16 to 18 min (5% A → 80% A); isocratic elution from 18 to 20 min (80% A). Solvent A consisted of 0.1% HCOOH in water, and solvent B consisted of 0.1% HCOOH in MeOH. The column temperature was 28 °C. The amounts of quercetin and its glycosides in the extracts after completing the biotransformation process were determined using standard calibration curves and using the peak area relationship between quercetin and its metabolites (detection at wavelength λ = 370 nm). The individual standard stock solutions of quercetin (purity (HPLC) ≥ 99%) (Extrasynthese, Genay, France), 3-*O*-glucoside of quercetin (purity (HPLC) ≥ 98%) (Extrasynthese, Genay, France) and 7-*O*-glucoside of quercetin (purity (HPLC) ≥ 99%) (Extrasynthese, Genay, France) were diluted to a series of different concentration solutions for constructing the calibration curves in the concentration range 181.0–905.0 µg/mL for quercetin, 137.5–687.5 µg/mL for 3-*O*-glucoside of quercetin and 128–320 µg/mL for 7-*O*-glucoside of quercetin. The mixtures of the standard solutions were injected in triplicate (5 µL), and the calibration curves were constructed by plotting the peak area (Y-axis) versus the concentration (X-axis) of each analyte. The results of all tested compounds showed good linearity: *R*^2^ = 0.9964 for quercetin, *R*^2^ = 0.9993 for 3-*O*-glucoside of quercetin and *R*^2^ = 0.9923 for 7-*O*-glucoside of quercetin. Data acquisition and analysis were performed using the Chromeleon software workstation (v7.2.10) (Thermo Scientific, Waltham, MA, USA).

### 3.6. Pharmacokinetics, Drug Nature, Biological Activity Prediction

The predictions of pharmacokinetic and physicochemical properties, medicinal chemistry friendliness and potential biological activity of flavonoid derivatives based on their structural formulae were computed using SwissADME (Available online: http://www.swissadme.ch (accessed on 30 May 2023)) and Way2Drug Pass Online with accompanying services (Available online: http://www.way2drug.com/PASSOnline (accessed on 30 May 2023)). The structures of the molecules were built with ACD Chemsketch 2021.2.0 and saved in a .mol format and, in this form, imported into both services. The biological activity types in Pass Online are shown as the probability to be revealed (Pa) and not to be revealed (Pi), and they are independent values in the range from 0 to 1.

### 3.7. Statistical Analysis

The HPLC conversion data in Supplementary Materials are presented as the mean ± standard error of the mean (SD). Statistical analysis was performed with one-way ANOVA with Tukey’s post hoc test (Excel Office ver. 2019 with Real Statistics Resource Pack, Microsoft, Redmond, WA, USA). The significance was accepted at a *p* value < 0.05.

## 4. Conclusions

Glycosylation is an effective way of improving the water solubility of natural products. Flavonoid glycosylation has been increasingly described in recent times, which does not necessarily mean we fully understand all the processes and enzymes responsible for these reactions.

In the presented paper, we described the capacity of entomopathogenic fungi strains to produce both 4-*O*-methylglucosides and a glucoside of quercetin.

Additionally, research on the properties of flavonoids, including their glycoside derivatives, is ongoing, and efficient methods for obtaining such compounds are sought continuously. The methods presented in this publication allow significant amounts of glycoside derivatives to be obtained efficiently and relatively cheaply while following the principles of “green chemistry”.

In vivo, in vitro and clinical studies remain essential for a comprehensive understanding of the biological activities, cytotoxicity and molecular mechanisms of action of potential novel drugs. However, modern predictive tools can play a significant role in accelerating the screening process for lead compounds. These tools, which include predicting pharmacokinetic parameters, such as bioavailability and absorption, as well as pharmacodynamic parameters for assessing therapeutic potential, could shorten the time and streamline the search for bioactive scaffolds. Identifying these pharmacophores is crucial in the development of new drugs.

Our research indicated variations in the bioavailability of quercetin and its glycosides when comparing in silico simulations with in vivo studies. These discrepancies clearly indicate that the algorithms used in the cheminformatics tools for predicting pharmacokinetic properties require further refinement.

## Figures and Tables

**Table 1:** Microbial transformation of quercetin, HPLC conversion.

Strain	Time of Biotransformation [Days]	Conversion [%] after 1, 3, 7 and 10 Days of Biotransformation				
		Substrate	Products			Other Products
		1	2	3	4	
*Beauveria bassiana* KCh J1.5	1	64	36	-	-	-
	3	30	70	-	-	-
	7	4	96	-	-	-
	10	1	99	-	-	-
*Beauveria bassiana* KCh BBT	1	68	32	-	-	-
	3	44	55	-	-	-
	7	32	68	-	-	-
	10	28	72	-	-	-
*Beauveria bassiana* KCh J3.2	1	95	-	-	-	5
	3	1	4	-	-	95
*Beauveria bassiana* KCh J2.1	10	91	2	-	-	7
*Beauveria bassiana* KCh J1	10	95	-	-	-	5
*Beauveria caledonica* KCh J3.3	1	97	3	-	-	-
	3	80	20	-	-	-
	7	68	32	-	-	-
	10	50	13	-	-	37
*Beauveria caledonica* KCh J3.4	10	99	-	-	-	1
*Isaria farinosa* KCh KW 1.1	1	96	0.4	0.1	0.6	3
	3	91	1.5	0.8	1.9	4
	7	86	2.9	1.5	3.5	6
	10	79	2.8	0.9	3.5	13
*Isaria tenuipes* MU35	1	80	1.7	2	16	-
	3	61	2.6	3.3	33	-
	7	13	4	7	70	-
	10	10	6	12	67	4
*Isaria tenuipes* CYS30	1	81	1.5	3.2	13	-
	3	43	4.2	7	43	-
	7	19	6	10	62	3
	10	6	7	13	69	5
*Metapochonia bulbillosa* CYS17	10	96	-	-	-	4
*Beauveria feline* ENC3	10	99	-	-	-	1
*Lecanicillium lecanii* DSM 63098	10	99	-	-	-	1
*Lecanicillium lecanii* NK3	10	99	-	-	-	1
*Metarhizium anisopliae* MU4	10	10	-	-	4	86

Data are expressed as the mean of three independent experiments (biological replicates). The table containing data expressed as means ± standard deviations can be found in the Supplementary Materials—Table S2a,b. **1**—Quercetin; **2**—(7-*O*-β-D-(4″-*O*-methylglucopyranosyl)-quercetin; **3**—3-*O*-β-D-(4″-*O*-methylglucopyranosyl)-quercetin; **4**—3-*O*-β-D-(glucopyranosyl)-quercetin; Other products—products of degradation. Selected chromatograms showing the progress of quercetin (**1**) biotransformation in the cultures of entomopathogenic filamentous fungi are available in Supplementary Data (Figure S23).

**Table 2:** Pharmacokinetic and pharmacodynamic data compiled by the online tool SwissADME.

Activity/Parameter	1	2	3	4
Lipophilicity (Consensus Log Po/w)	1.23	−0.02	0.00	−0.48
Water solubility	0.17 mg/mL; 5.73 × 10^−4^ mol/L	3.03 mg/mL; 6.32 × 10^−3^ mol/L	3.03 mg/mL; 6.32 × 10^−3^ mol/L	14.30 mg/mL; 3.08 × 10^−2^ mol/L
Gastrointestinal absorption	High	Low	Low	Low
BBB permeant	No	No	No	No
P-gp substrate	No	No	No	No
CYP1A2 inhibitor	Yes	No	No	No
CYP2C19 inhibitor	No	No	No	No
CYP2C9 inhibitor	No	No	No	No
CYP2D6 inhibitor	Yes	No	No	No
CYP3A4 inhibitor	Yes	No	No	No
Log Kp (skin permeation)	−7.05 cm/s	−8.97 cm/s	−8.97 cm/s	−8.88 cm/s
Bioavailability score	0.55	0.17	0.17	0.17

**Table 3:** Biological activity predictions for quercetin (**1**) and its metabolites (**2, 3, 4**) using the passOnline tool.

Activity	1		2		3		4	
	Pa	Pi	Pa	Pi	Pa	Pi	Pa	Pi
Membrane permeability inhibitor	0.938	0.003	0.978	0.001	0.979	0.001	0.981	0.001
Membrane integrity agonist	0.973	0.002	0.977	0.002	0.983	0.001	0.989	0.001
Hemostatic	0.771	0.003	0.977	0.001	0.979	0.001	0.987	0.001
Free radical scavenger	0.811	0.003	0.973	0.001	0.978	0.001	0.978	0.001
Monophenol monooxygenase inhibitor	0.792	0.003	0.970	0.001	0.981	0.001	0.983	0.001
Cardioprotectant	0.833	0.003	0.968	0.001	0.974	0.001	0.984	0.001
Anticarcinogenic	0.757	0.007	0.961	0.001	0.969	0.001	0.965	0.001
Hepatoprotectant	0.706	0.007	0.955	0.002	0.964	0.001	0.961	0.001
Chemopreventive	0.717	0.006	0.949	0.002	0.954	0.002	0.956	0.002
Vasoprotector	0.824	0.004	0.948	0.002	0.934	0.002	0.947	0.002
UDP-glucuronosyltransferase substrate	0.857	0.004	0.936	0.003	0.906	0.003	0.954	0.002
Lipid peroxidase inhibitor	0.788	0.004	0.934	0.002	0.943	0.002	0.976	0.002
Proliferative diseases treatment	0.614	0.010	0.913	0.002	0.929	0.002	0.921	0.002
Capillary fragility treatment	0.737	0.001	0.882	0.000	0.888	0.000	0.913	0.000
Antioxidant	0.872	0.003	0.846	0.003	0.877	0.003	0.913	0.003
Antimutagenic	0.940	0.001	0.720	0.005	0.714	0.006	0.763	0.004

Pa—probable activity; Pi—probable inactivity. Values range from 0 to 1, where 1 represents 100% probability of Pa or Pi, and 0 represents 0% probability of Pa or Pi.

**Table 4:** Identification of fungal strains on the basis of the sequence of the ITS1-ITS2 sequences and comparison with reference ITS sequences.

Name of Fungal Strain	Identified Fungal Species	Sequence Identity	Accession Numbers
CYS17	*Metapochonia bulbillosa*	99% identity with *Metapochonia bulbillosa*,	Acc. Numbers: OK661050.1, DQ132810.1, MK164206.1
CYS30	*Isaria tenuipes*	100% identity with *Isaria tenuipes*,	Acc. Numbers: MT966070.1, MT966058.1, MT966055.1
MU35	*Isaria tenuipes*	100% identity with *Isaria tenuipes*,	Acc. Numbers: MT966070.1, MT966058.1, MT966055.1
MU4	*Metarhizium anisopliae*	100% identity with *Metarhizium anisopliae*,	Acc. Numbers: FJ177507.1, and 99%: FJ177475.1, EU307928.1

## Data Availability

Samples of the compounds **1**–**4** are available from the authors.

## Supplementary Materials

The following supporting information can be downloaded at: https://www.mdpi.com/article/10.3390/ijms241411857/s1.
