# Supplementary material for: Glycosylation of Quercetin by Selected Entomopathogenic Filamentous Fungi and Prediction of Its Products’ Bioactivity
Source: Int J Mol Sci. 2023 Jul 24;24(14):11857. doi: 10.3390/ijms241411857 (PMC10380404; doi:10.3390/ijms241411857)
Supplement: Supplementary file 1 [file ijms-24-11857-s001.zip › ijms-2517348-supplementary.pdf]

Supplementary Data

Glycosylation of quercetin by selected entomopathogenic filamentous fungi and prediction of its products' bioactivity

Tomasz Tronina[1]*, Mateusz Łużny[1], Monika Dymarska[1], Monika Urbaniak[2], Ewa Kozłowska[1], Michał Piegza[3], Łukasz Stępień[2] and Tomasz Janeczko[1]*

[1] Department of Food Chemistry and Biocatalysis, Wrocław University of Environmental and Life Sciences, Norwida 25, 50-375 Wrocław, Poland; mat.luzny@gmail.com (M.Ł.); monika.dymarska@gmail.com (M.D.); e.a.kozlowska@gmail.com (E.K.)

[2] Department of Pathogen Genetics and Plant Resistance, Institute of Plant Genetics, Polish Academy of Sciences, Strzeszyńska 34, 60-479 Poznań, Poland; murb@igr.poznan.pl (MU); lste@igr.poznan.pl (ŁS)

[3] Department of Biotechnology and Food Microbiology, Wrocław University of Environmental and Life Sciences, Chełmońskiego 37, 51-630 Wrocław, Poland; michal.piegza@upwr.edu.pl (MP)

* Correspondence: tomasz.tronina@upwr.edu.pl (T.T.); janeczko13@interia.pl (T.J.); Tel.: +48-713-205-195

Content

Table S1. Identification of fungal strains on the basis of the sequence of the ITS1-ITS2 sequences and comparison with reference ITS sequences.

Figure S1. Structure of quercetin (**1**)

Figure S2. UV spectra of quercetin (**1**)

Supplementary Data 1. NMR Spectral data of quercetin (**1**)

Figure S3. ^{1}H NMR spectra of quercetin (**1**) (600MHz, DMSO-d_6)

Figure S4. ^{13}C NMR spectra of quercetin (**1**) (151 MHz, DMSO-d_6)

Figure S5. Structure of 7-*O*-β-D-(4″-O-methylglucopyranosyl)-quercetin (**2**)

Figure S6. UV spectra of 7-*O*-β-D-(4″-O-methylglucopyranosyl)-quercetin (**2**)

Supplementary Data 2. NMR Spectral data of 7-*O*-β-D-(4″-O-methylglucopyranosyl)-quercetin (**2**)

Figure S7. ^{1}H NMR spectra of 7-*O*-β-D-(4″-O-methylglucopyranosyl)-quercetin (**2**) (600MHz, DMSO-d_6)

Figure S8. ^{13}C NMR spectra of 7-*O*-β-D-(4″-O-methylglucopyranosyl)-quercetin (**2**) (151 MHz, DMSO-d_6)

Figure S9. ^{1}H -^{1}H NMR (COSY) spectrum of quercetin 7-O-β-D-(4''-O-methyl)glucopyranoside (**2**) (600 /600 MHz, DMSO-d_6)

Figure S10. ^{1}H -^{13}C NMR (HSQC) spectrum of quercetin 7-O-β-D-(4''-O-methyl)glucopyranoside (**2**) (600 /151 MHz, DMSO-d_6)

Figure S11. Structure of 3-*O*-β-D-(4″-O-methylglucopyranosyl)-quercetin (**3**)

Figure S12. UV spectra of 3-*O*-β-D-(4″-O-methylglucopyranosyl)-quercetin (**3**)

Supplementary Data 3. NMR Spectral data of 3-*O*-β-D-(4″-O-methylglucopyranosyl)-quercetin (**3**)

Figure S13. ^{1}H NMR spectra of 3-*O*-β-D-(4″-O-methylglucopyranosyl)-quercetin (**3**) (600MHz, Acetone-d_6)

Figure S14. ^{13}C NMR spectra of 3-*O*-β-D-(4″-O-methylglucopyranosyl)-quercetin (**3**) (151 MHz, Acetone-d_6)

Figure S15. ^{1}H -^{1}H NMR (COSY) spectrum of quercetin 3-*O*-β-D-(4″-O-methylglucopyranosyl)-quercetin (**3**) (600/600 MHz, Acetone-d_6)

Figure S16. ^{1}H -^{13}C NMR (HSQC) spectrum of quercetin 3-*O*-β-D-(4″-O-methylglucopyranosyl)-quercetin (**3**) (600 /151 MHz, Acetone-d_6)

Figure S17. Structure of 3-*O*-β-D-(glucopyranosyl)-quercetin (**4**)

Figure S18. UV spectra of 3-*O*-β-D-(glucopyranosyl)-quercetin (**4**)

Supplementary Data 4. NMR Spectral data of 3-*O*-β-D-(glucopyranosyl)-quercetin (**4**)

Figure S19. ^{1}H NMR spectra of 3-*O*-β-D-(glucopyranosyl)-quercetin (**4**) (600MHz, Acetone-d_6)

Figure S20. ^{13}C NMR spectra of 3-*O*-β-D-(glucopyranosyl)-quercetin (**4**) (151 MHz, Acetone-d_6)

Figure S21. ^{1}H -^{1}H NMR (COSY) spectrum of quercetin 3-*O*-β-D-(glucopyranosyl)-quercetin (**4**) (600/600 MHz, Acetone-d_6)

Figure S22. ^{1}H -^{13}C NMR (HSQC) spectrum of quercetin 3-*O*-β-D-(glucopyranosyl)-quercetin (**4**) (600/151 MHz, Acetone-d_6)

Figure S23. Selected chromatograms showing progress of quercetin (**1**) biotransformation in the cultures of entomopathogenic filamentous fungi.

Table S2a. Microbial transformation of quercetin, HPLC conversion expressed by means and standard deviation.

Table S2b. Microbial transformation of quercetin, HPLC conversion expressed by means and standard deviation (continuation).

Figure S24. Progress in production of compound **2** by entomopathogenic fungi.

Figure S25. Progress in production of compound **3** by entomopathogenic fungi.

Figure S26. Progress in production of compound **4** by entomopathogenic fungi.

Table S1. Identification of fungal strains on the basis of the sequence of the ITS1-ITS2 sequences and comparison with reference ITS sequences.

Name of fungal strain	Identified fungal species	Sequence identity	Sequence
CYS17	*Metapochonia bulbillosa*	99% identity to *Metapochonia bulbillosa*, Acc. Numbers: OK661050.1, DQ132810.1, MK164206.1	GGTCACTCTAAAAAGTTGGGCGTTTTACGGCAGTGGCCGCGTCGCG<br>CTCccgctgcgagGTTGTGCTACTACGCAGAGGAGGCCGCGACGgggccg<br>CCAATTCATTTCGGGGGCGGCGGACGCGCCGGGGGTGTTCCCCCGG<br>GCGAGGTCGCCGGTCCCCAACACCAGGCCACTGGGGCTTGAGGGTT<br>GAAATGACGCTCGAACAGGCATGCCCGCCAGAATACTGGCGGGCGC<br>AATGTGCGTTCAAAGATTCGATGATTCACTGAATTCTGCAATTCACAT<br>TACTTATCGCATTTCGCTGCGTTCTTCATCGATGCCAGAACCAAGAGA<br>TCCGTTGTTGAAAGTTTTGATTCATTTGTTTATGATTCCACTCAGACAT<br>GCTATAAAAAAGATACAAGAGTTTTGGTCCCCCGGCGGGCGCCTGG<br>TTCCGGCGGCCCTCGCGGGCCTTCCGGGGCGTTaACCCGCCGAAGC<br>AACAGTAAAGGTATAAGTTCACAGGGGTTTGGGAGTAGAATAACTC<br>GGTAATGATCCCTCCGC
CYS30	*Isaria tenuipes*	100% identity to *Isaria tenuipes*, Acc. Numbers: MT966070.1, MT966058.1, MT966055.1	GTCAcGTTCAGAgGTTGGGGGTTTCACGGCGGGCCGCGTCGGGTTCC<br>cgGtgcgaGTGCTTGTACTGCGCAGAGGTCGCCGCGGACGGGCCgCCA<br>CTCCATTTCAGGGCCGGCGGGGTGCTGCCGGTCCCCAAGGCCGACG<br>TCCCGGGGGACGTCGAGGGTTGAAATGACGCTCGAACAGGCATGCC<br>CGCCAGAATGCTGGCGGGCGCAATGTGCGTTCAAAGATTCGATGAT<br>TCACGGAATTCTGCAATTCACATTACGTATCGCATTTCGCTGCGTTCT<br>TCATCGATGCCAGAACCAAGAGATCCGTTGTTGAAAGTTTTGATTCG<br>TTTGTGTTGCCTTGCGGCGGATTCAGAGAGGCTGACAGATACAGGG<br>TTGCGTGGTCCCCGGCGGCCGCCTGGGTCCAGGTCGCGGGCCGGCG<br>CTGGGCCGTCCGGACGCTGGGGCGGGTCCGCCGAAGCAACTATGG<br>GTAGGTTCACAGAAGGGTTGGGAGTTGTAAAACTCTGGTAATGATC<br>CCTCCG
MU35	*Isaria tenuipes*	100% identity to *Isaria tenuipes*, Acc. Numbers: MT966070.1, MT966058.1, MT966055.1	GTCACGTTCAGAgGTTGGGGGTTTCACGGCGGGCCGCGTCGGGTTCc<br>cggtgcgaGTGCTTGTACTGCGCAGAGGTCGCCGCGGACGGGCCGCCA<br>CTCCATTTCAGGGCCGGCGGGGTGCTGCCGGTCCCCAAGGCCGACG<br>TCCCGGGGGACGTCGAGGGTTGAAATGACGCTCGAACAGGCATGCC<br>CGCCAGAATGCTGGCGGGCGCAATGTGCGTTCAAAGATTCGATGAT<br>TCACGGAATTCTGCAATTCACATTACGTATCGCATTTCGCTGCGTTCT<br>TCATCGATGCCAGAACCAAGAGATCCGTTGTTGAAAGTTTTGATTCG<br>TTTGTGTTGCCTTGCGGCGGATTCAGAGAGGCTGACAGATACAGGG<br>TTGCGTGGTCCCCGGCGGCCGCCTGGGTCCAGGTCGCGGGCCGGCG<br>CTGGGCCGTCCGGACGCTGGGGCGGGTCCGCCGAAGCAACTATGG<br>GTAGGTTCACAGAAGGGTTGGGAGTTGTAAAACTCTGGTAATGATC<br>CCTCCGCAG
MU4	*Metarhizium anisopliae*	100% identity to *Metarhizium anisopliae*, Acc. Numbers: FJ177507.1, and 99%: FJ177475.1, EU307928.1	GTCACTATAAAaGTTGGGGGGTTTTACGGCAGTGGacCGCGCCGGG<br>CTCCtgttGCGaGTGTTTTACTACTGCGCAGAGGAGGGCCACGGCgag<br>ACCGCCAATTGATTTGAGGGACGGCTGCGCTGGAAAACCAGCCTCG<br>CCGATCCCCAACACCAAGTCCACAGGGGACTTGAGGGGCGTAATGA<br>CGCTCGAACAGGCATGCCCGCCAGAATACTGACGGGCGCAATGTGC<br>GTTCAAAGATTCGATGATTCACTGAATTCTGCAATTCACATTACTTAT<br>CGCATTTCGCTGCGTTCTTCATCGATGCCAGAACCAAGAGATCCGTT<br>GTTGAAAGTTTTGATTCATTTTTTTAACCACTCAGAAGATACTTATTA<br>AAAAATTCAGAAGGTTTGGGTCCCCGGCGGGCGCGAAGTCCCGCCG<br>AAGCAACAATTAAAGGTATAATTCACAGGGGTTGGGAGtTGGATAA<br>CTCGGTAATGATCCCTCCGCA

Figure S1. Structure of quercetin (**1**)

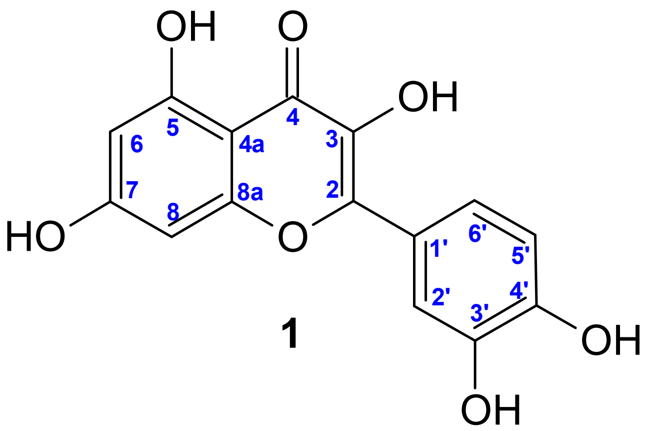

Figure S2. UV spectra of quercetin (**1**)

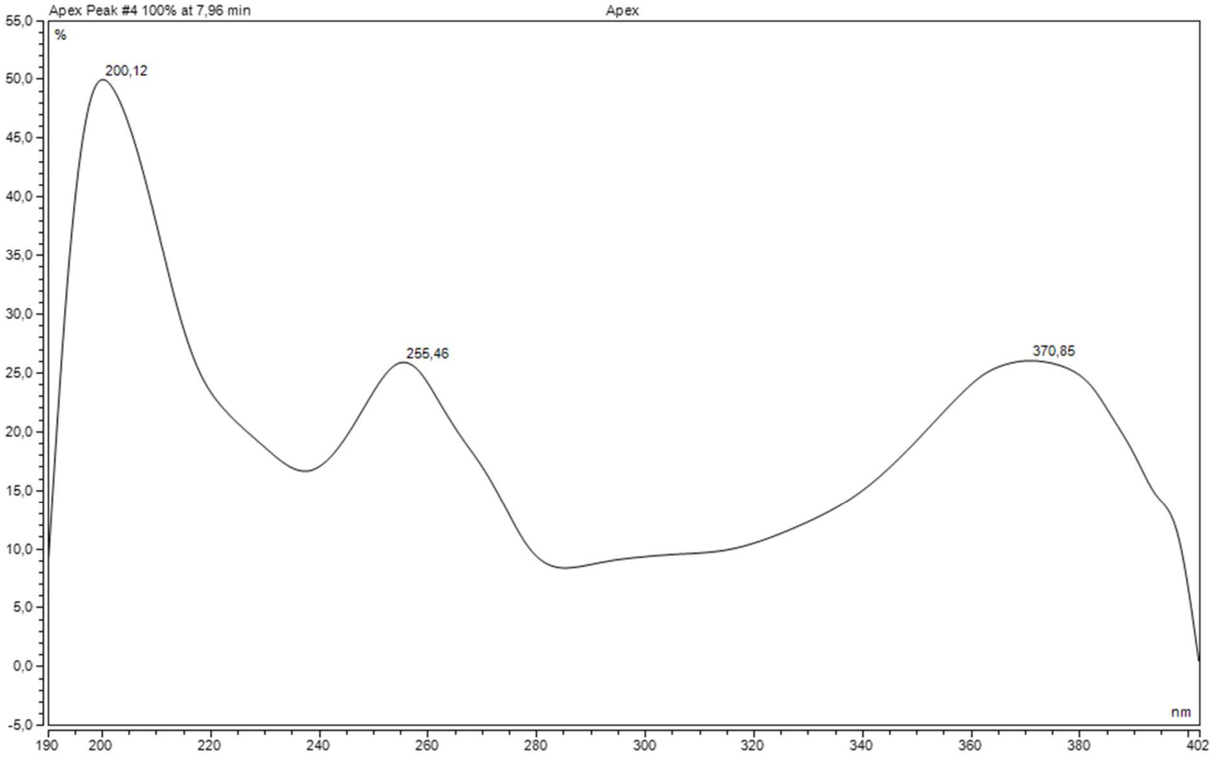

Supplementary Data 1. NMR Spectral data of quercetin (**1**): ^{1}H-NMR (DMSO-d_6) δ_H: 6.19 (1H, d, J = 2.1 Hz, H-6), 6.41 (1H, d, J = 2.1 Hz, H-8), 6.89 (1H, d, J = 8.5 Hz, H-5′), 7.54 (1H, dd, J = 8.5, 2.2 Hz, H-6′), 7.68 (1H, d, J = 2.2 Hz, H-2′). ^{13}C-NMR (DMSO-d_6) δ_C: 93.2 (C-8), 98.0 (C-6), 102.9 (C-10), 114.9 (C-2′), 115.4 (C-5′), 119.8 (C-6′), 121.8 (C-1′), 135.6 (C-3), 144.9 (C-3′), 146.6 (C-2), 147.5 (C-4′), 156.0 (C-9), 160.6 (C-5), 163.7 (C-7), 175.7 (C-4).

Figure S3. ^{1}H NMR spectra of quercetin (**1**) (600MHz, DMSO-*d*6)

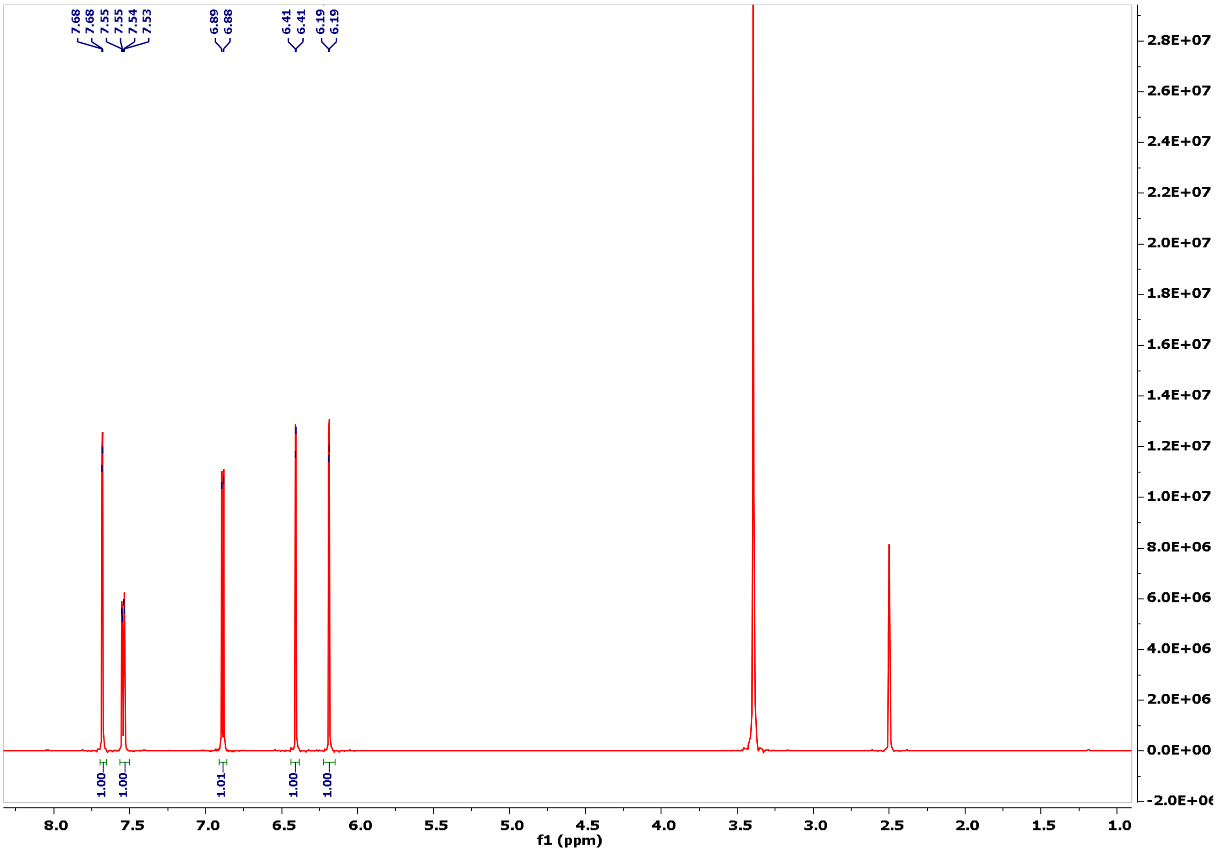

Figure S4. ^{13}C NMR spectra of quercetin (**1**) (151 MHz, DMSO-*d*6)

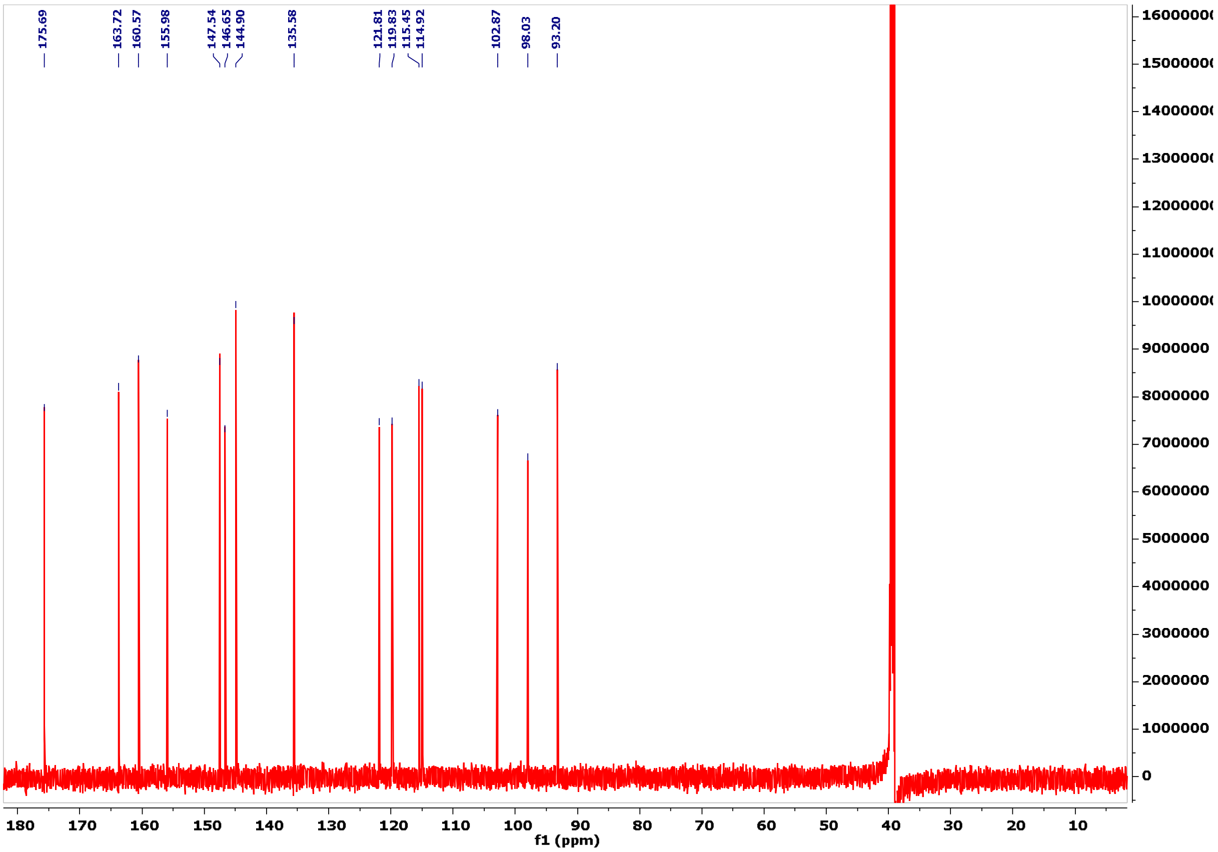

Figure S5. Structure of 7-*O*-β-D-(4″-*O*-methylglucopyranosyl)-quercetin (**2**)

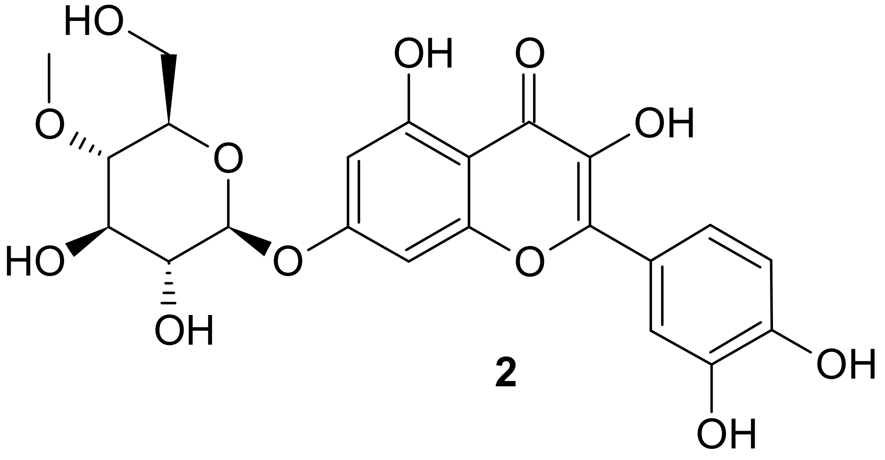

Figure S6. UV spectra of 7-*O*-β-D-(4″-*O*-methylglucopyranosyl)-quercetin (**2**)

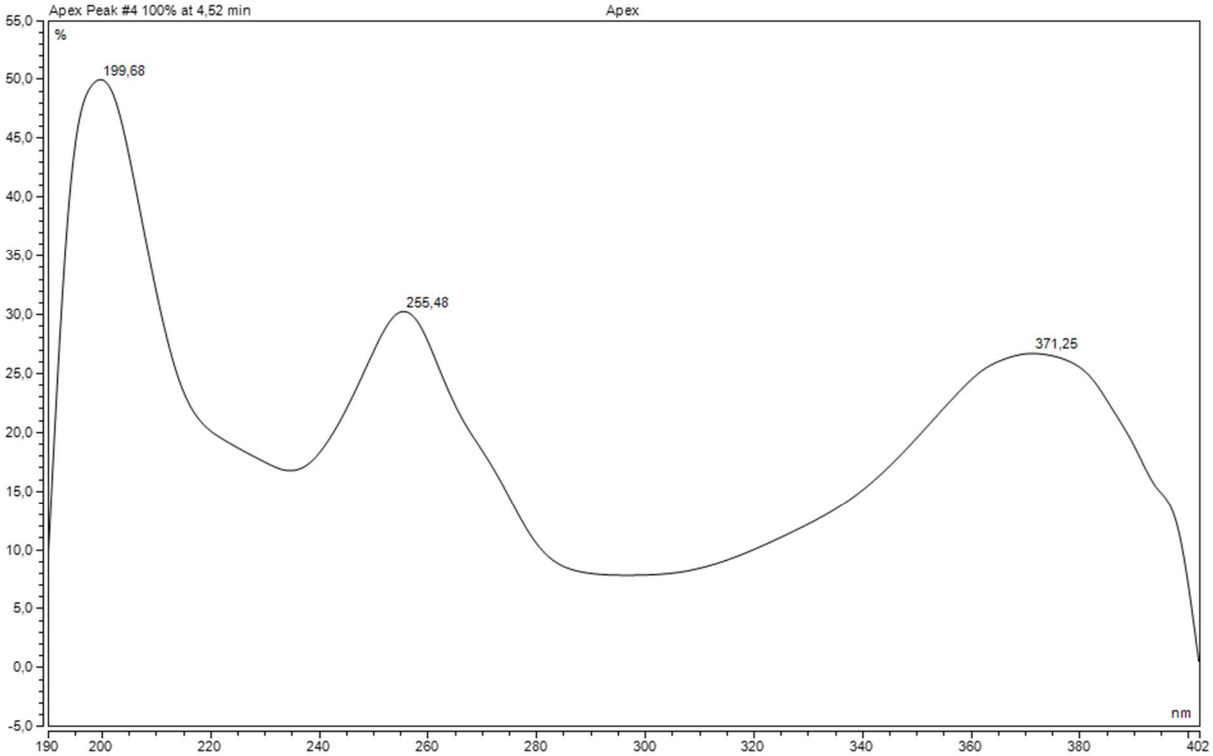

Supplementary Data 2. NMR Spectral data of 7-*O*-β-D-(4″-*O*-methylglucopyranosyl)-quercetin (**2**): ^{1}H-NMR (DMSO-d_6) δH: 3.05 (1H, t, *J* = 9.0 Hz, H-4″), 3.26 (1H, dd, *J* = 9.0, 7.8 Hz, H-2″), 3.44 (1H, t, *J* = 9.0 Hz, H-3″), 3.46 (3H, s, C4″-OCH_3), 3.49–3.53 (1H, m, C-5″ overlapped on H-6″a), 3.49–3.53 (1H, m, H-1″a, overlapped on H-5″), 3.62–3.68 (1H, m, H-6″b), 5.10 (1H, d, *J* = 7.8 Hz, H-1″), 6.42 (1H, d, *J* = 2.2 Hz, H-6), 6.76 (1H, d, *J* = 2.2 Hz, H-8), 6.90 (1H, d, *J* = 8.5 Hz, H-5′), 7.55 (1H, dd, *J* = 8.5, 2.2 Hz, H-6′), 7.72 (1H, d, *J* = 2.2 Hz, H-2′). ^{13}C-NMR (DMSO-d_6) δC: 59.7 (C-4″-OCH_3), 60.1 (C-6″), 73.2 (C-2″), 75.6 (C-5″), 76.0 (C-3″), 78.8 (C-4″), 94.2 (C-8), 98.6 (C-6), 99.5 (C-1″), 104.6 (C-4a), 115.3 (C-2′), 115.5 (C-5′), 120.0 (C-6′), 121.8 (C-1′), 136.0 (C-3), 145.0 (C-3′), 147.5 (C-2), 147.8 (C-4′), 155.7 (C-8a), 160.1 (C-5) 162.6 (C-7), 175.9 (C-4).

Figure S7. ^{1}H NMR spectra of 7-*O*-β-D-(4″-*O*-methylglucopyranosyl)-quercetin (**2**) (600MHz, DMSO-*d*$_6$)

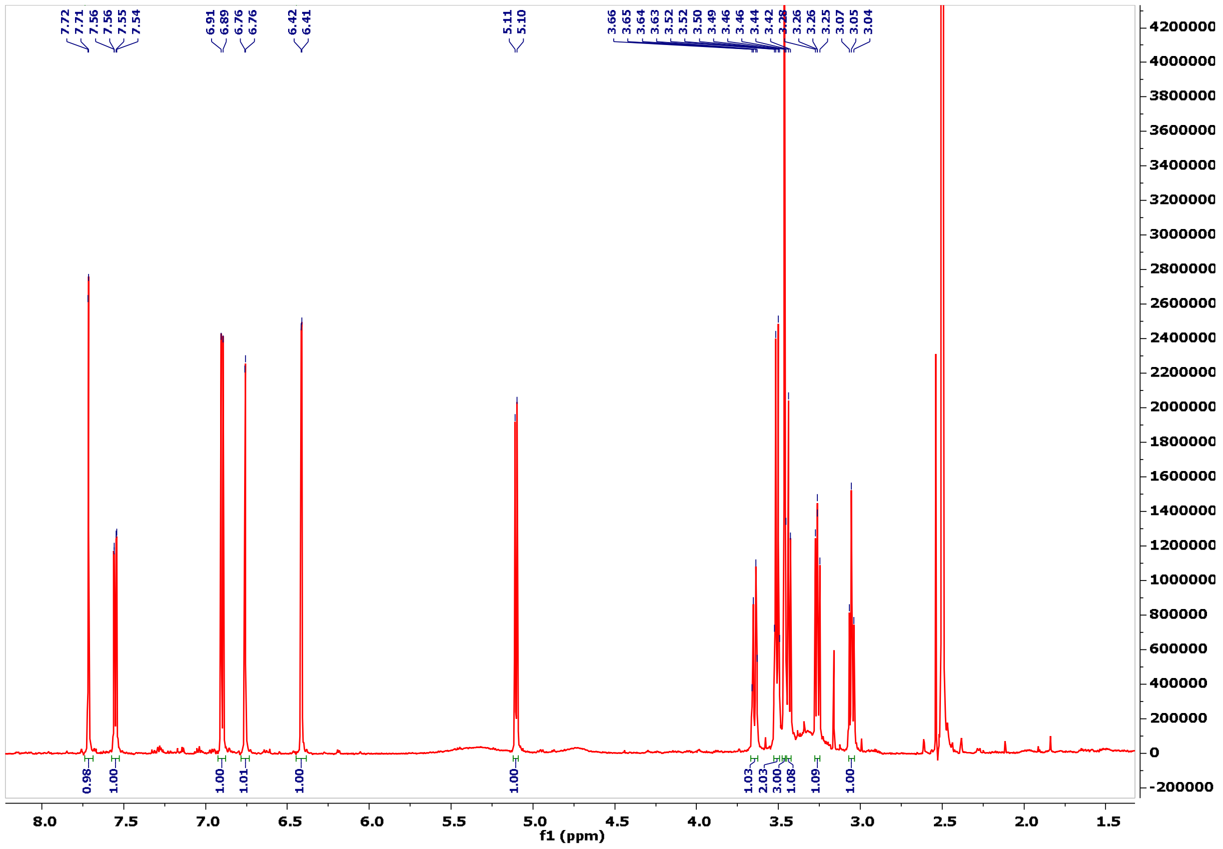

Figure S8. ^{13}C NMR spectra of 7-*O*-β-D-(4″-*O*-methylglucopyranosyl)-quercetin (**2**) (151 MHz, DMSO-*d*$_6$)

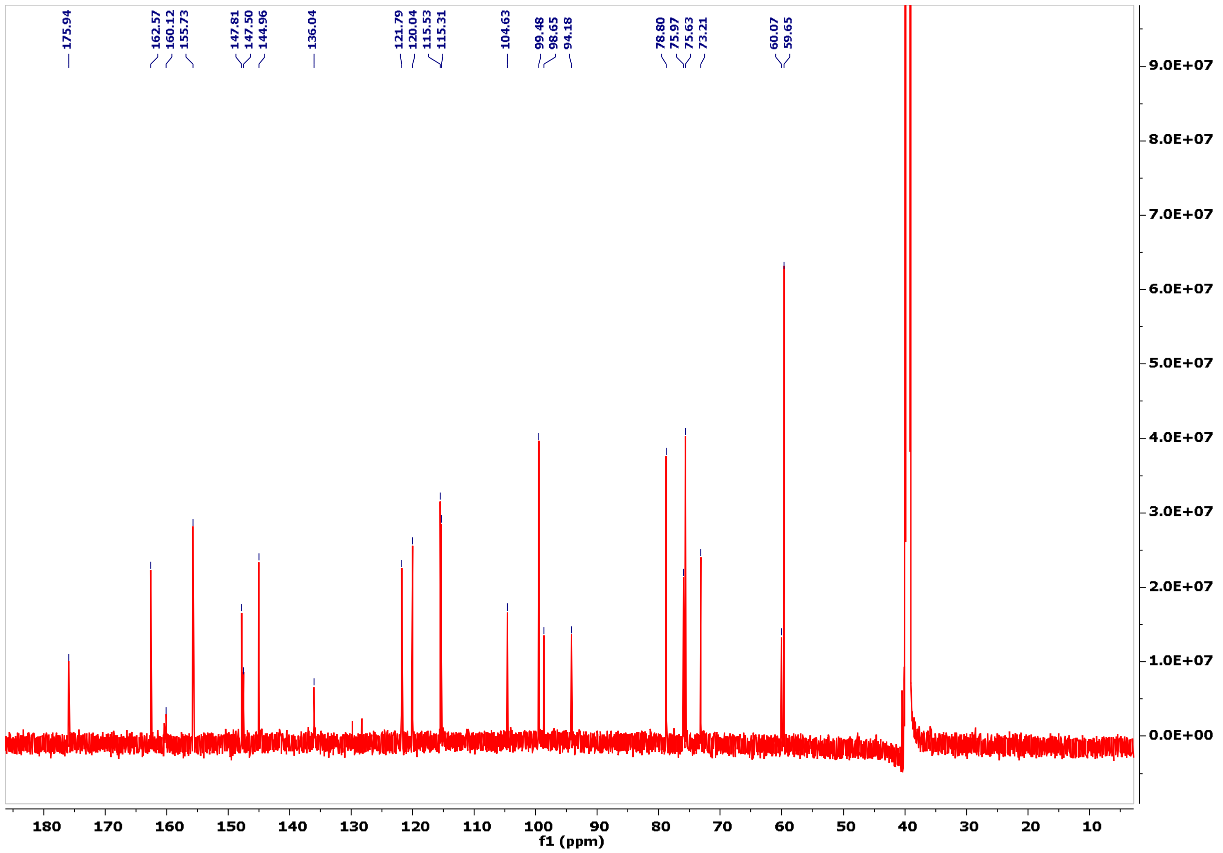

Figure S9. ^{1}H -^{1}H NMR (COSY) spectrum of quercetin 7-*O*-β-D-(4"-*O*-methyl)glucopyranoside (**2**) (600 /600 MHz, DMSO-d_6)

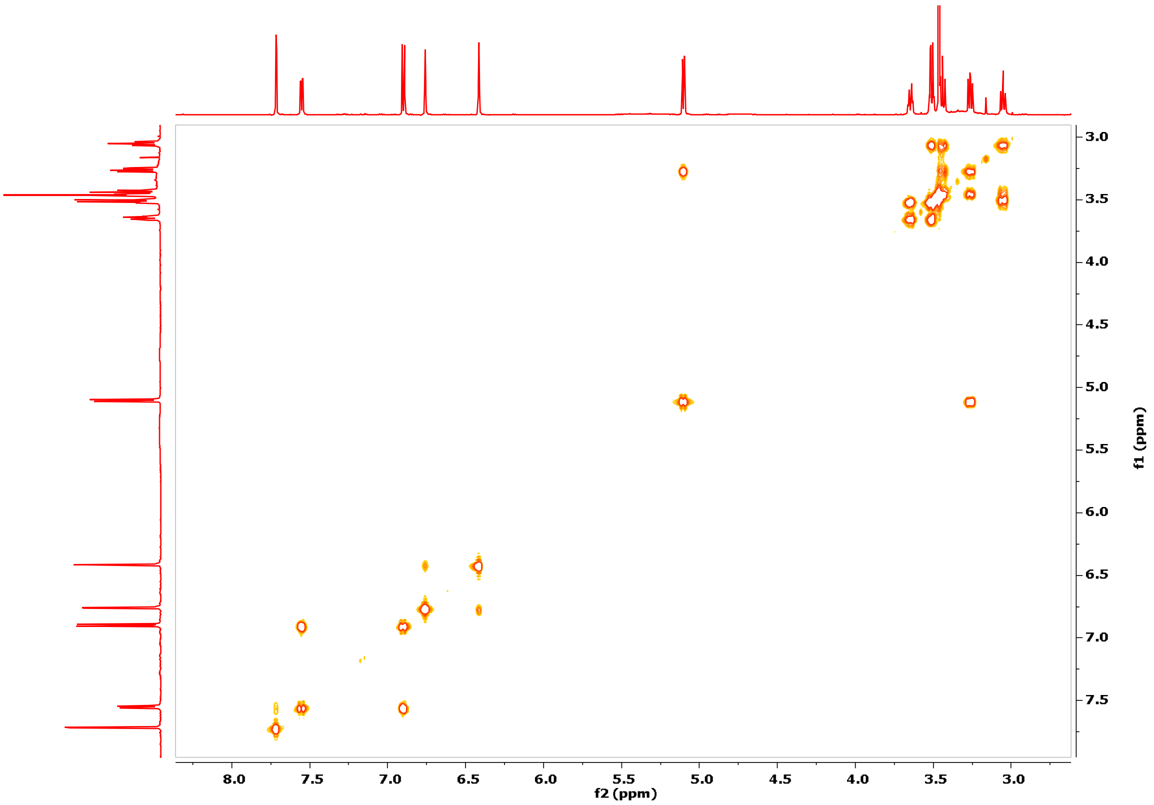

Figure S10. ^{1}H -^{13}C NMR (HSQC) spectrum of quercetin 7-*O*-β-D-(4"-*O*-methyl)glucopyranoside (**2**) (600 /151 MHz, DMSO-d_6)

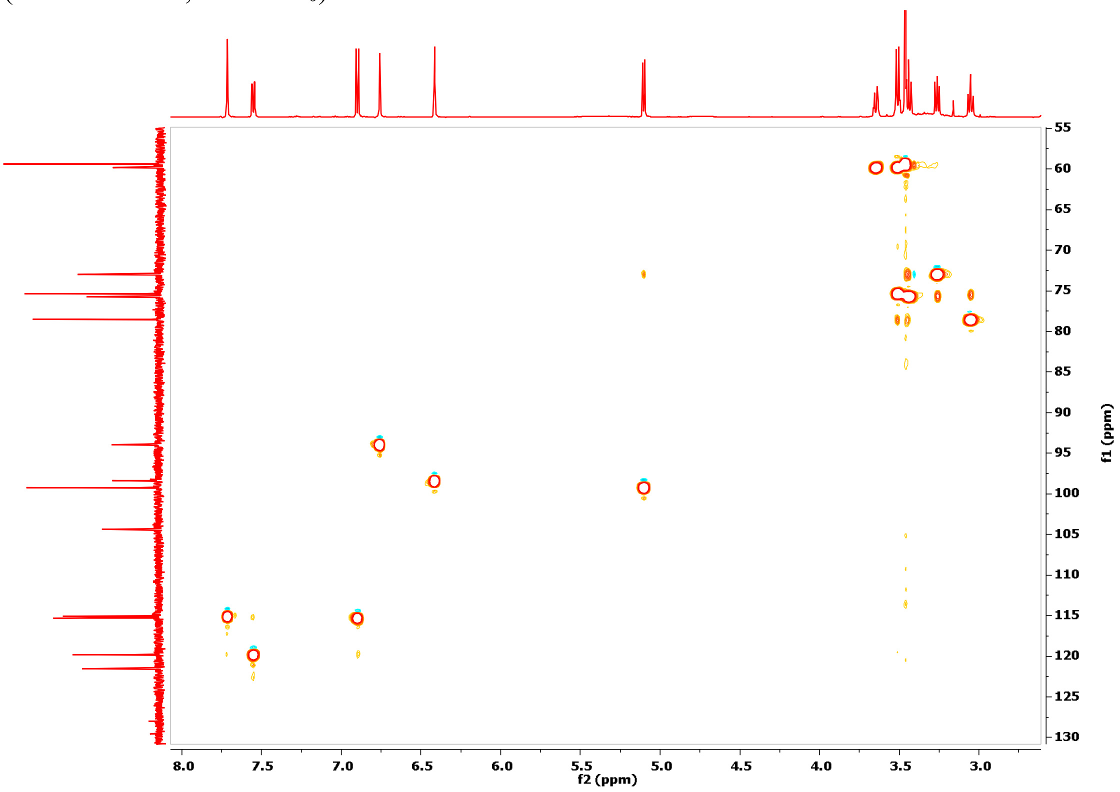

Figure S11. Structure of 3-*O*-β-D-(4″-*O*-methylglucopyranosyl)-quercetin (**3**)

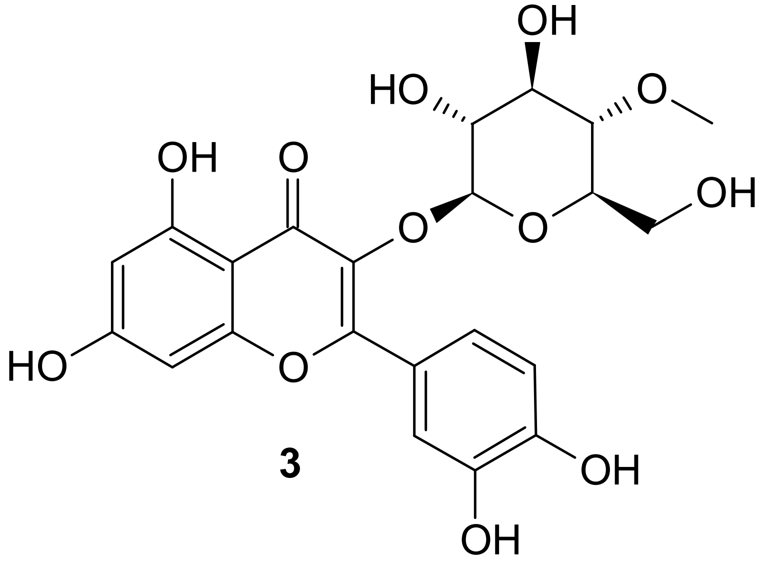

3

Figure S12. UV spectra of 3-*O*-β-D-(4″-*O*-methylglucopyranosyl)-quercetin (**3**)

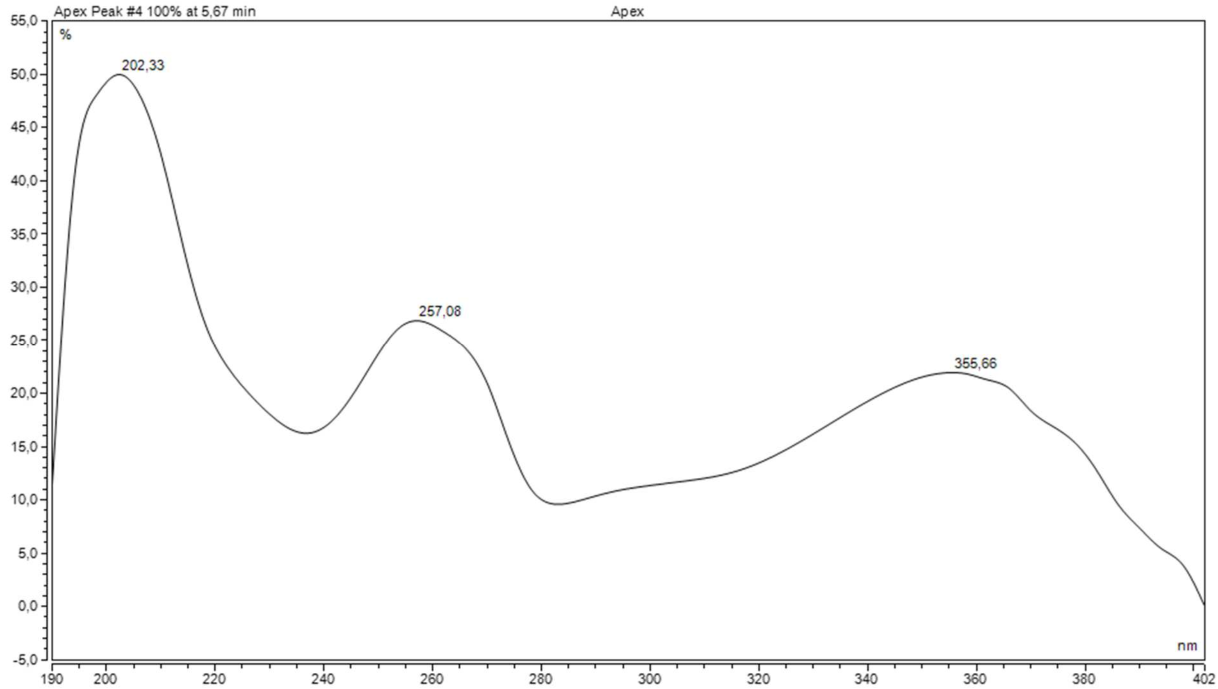

Supplementary Data 3. NMR Spectral data of 3-*O*-β-D-(4″-*O*-methylglucopyranosyl)-quercetin (**3**): ^{1}H-NMR (Acetone-d_6) δH: 3.**18** (1H, t, *J* = 9.0 Hz, H-4″), 3.34 (1H, ddd, *J* = 9.0, 5.0, 2.3 Hz, 5′-H), 3.50 (1H, t, *J* = 7.9 Hz, H-2″), 3.57 (3H, s, C4″-OCH_3), 3.60-3.66 (2H, m, H-3″ and one of H-6″), 3.73 (1H, dd, *J* = 12.0, 2.3 Hz, one of H-6″), 5.27 (1H, d, *J* = 7.9 Hz, H-1″), 6.32 (1H, d, *J* = 2.2 Hz, H-6), 6.55 (1H, d, *J* = 2.2 Hz, H-8), 7.00 (1H, d, *J* = 8.4 Hz, H-5′), 7.64 (1H, dd, *J* = 8.4, 2.2 Hz, H-6′), 8.04 (1H, d, *J* = 2.2 Hz, H-2′). ^{13}C-NMR (DMSO-d_6) δC: 60.5 (C-4″-OCH_3), 62.3 (C-6″), 75.7 (C-2″), 77.0 (C-5″), 78.2 (C-3″), 79.8 (C-4″), 94.7 (C-6), 99.8 (C-8), 104.8 (C-1″), 105.5 (C-4a), 115.9 (C-5′), 118.0 (C-2′), 122.7 (C-1′), 122.8 (C-6′), 135.6 (C-3), 145.3 (C-3′), 149.3 (C-4′), 158.0 (C-5), 158.8 (C-2), 162.9 (C-**8a**), 165.3 (C-7), 179.2 (C-4).

Figure S13. ^{1}H NMR spectra of 3-*O*-β-D-(4″-*O*-methylglucopyranosyl)-quercetin (**3**) (600MHz, Acetone-d_6)

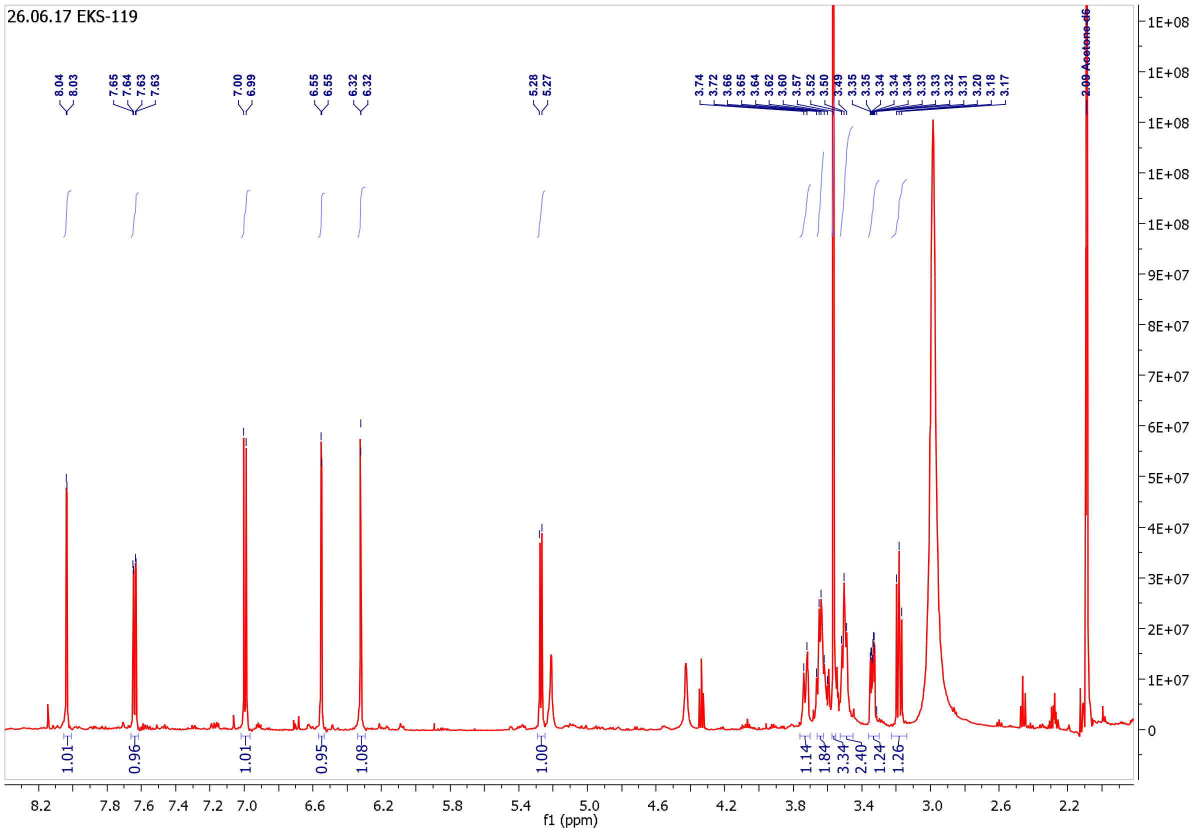

Figure S14. ^{13}C NMR spectra of 3-*O*-β-D-(4″-*O*-methylglucopyranosyl)-quercetin (**3**) (151 MHz, Acetone-d_6)

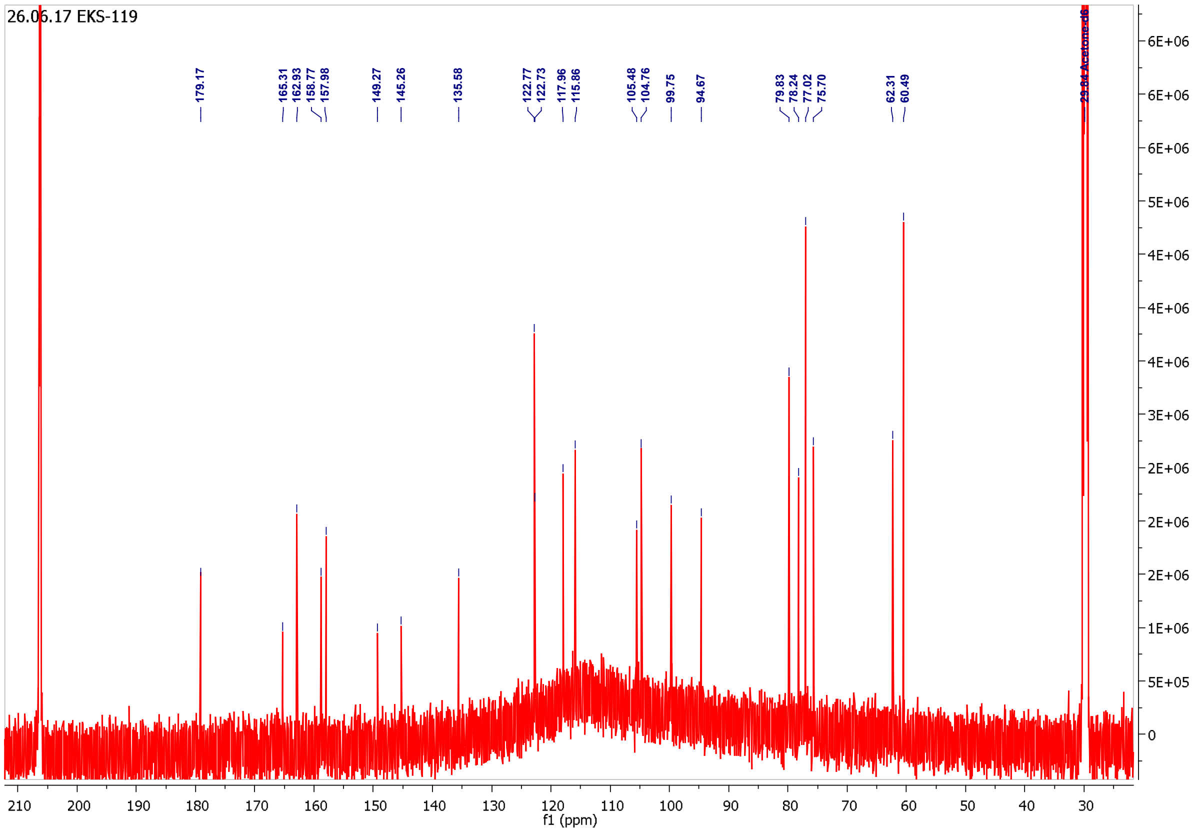

Figure S15. ^{1}H -^{1}H NMR (COSY) spectrum of quercetin 3-*O*-β-D-(4”-*O*-methylglucopyranosyl)-quercetin (**3**) (600 /600 MHz, Acetone-*d*$_6$)

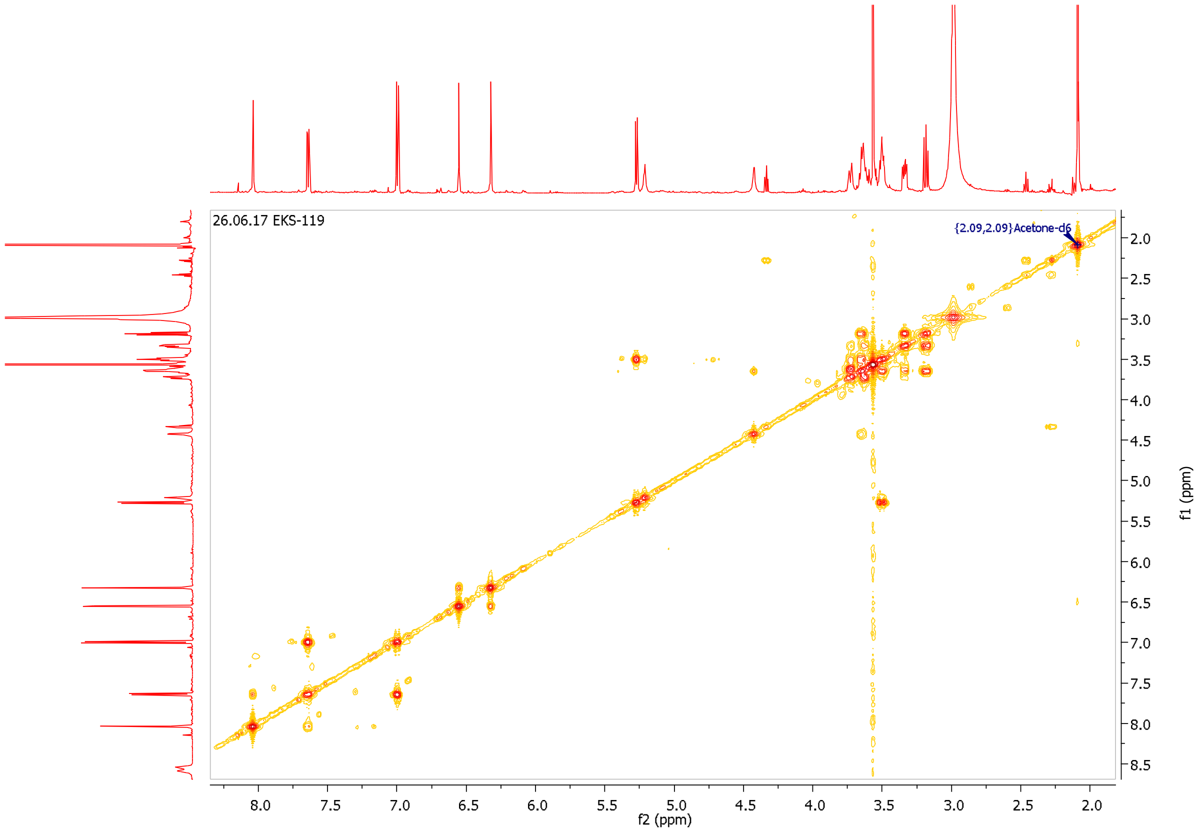

Figure S16. ^{1}H -^{13}C NMR (HSQC) spectrum of quercetin 3-*O*-β-D-(4”-*O*-methylglucopyranosyl)-quercetin (**3**) (600 /151 MHz, Acetone-*d*$_6$)

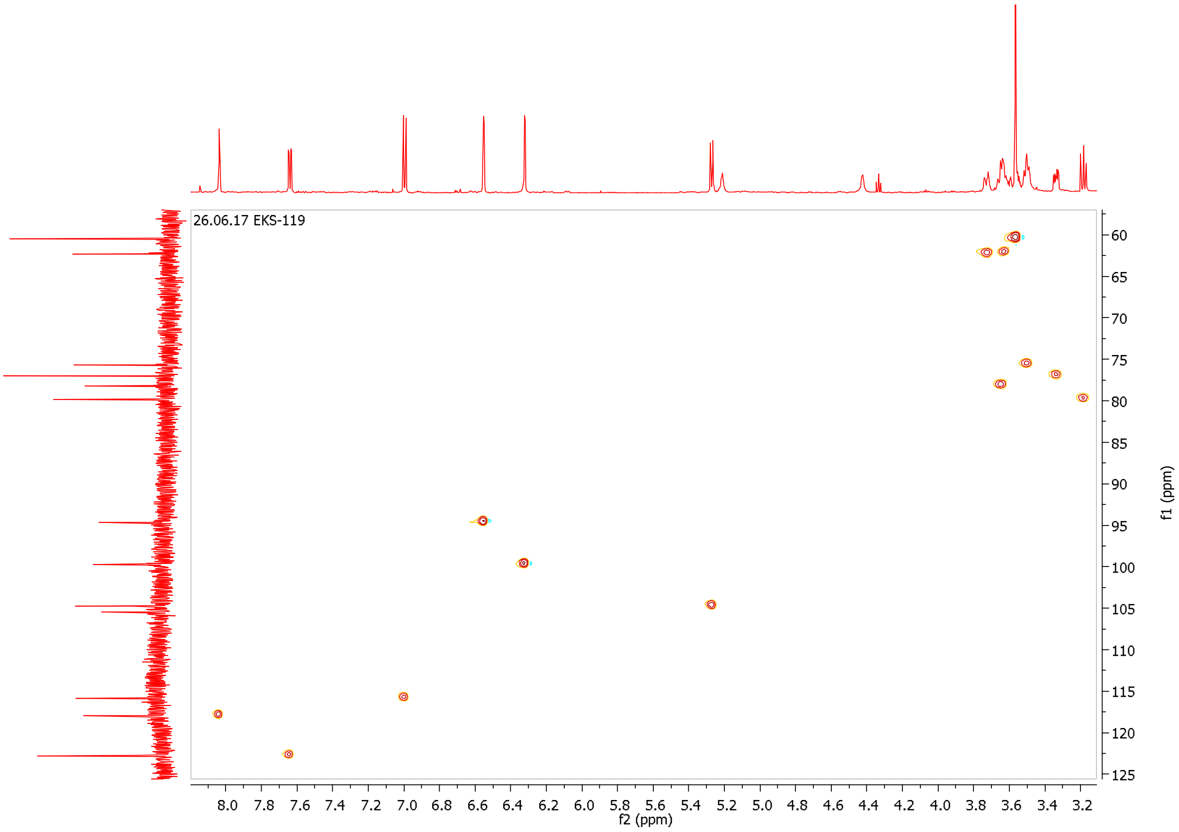

Figure S17. Structure of 3-*O*-β-D-(glucopyranosyl)-quercetin (**4**)

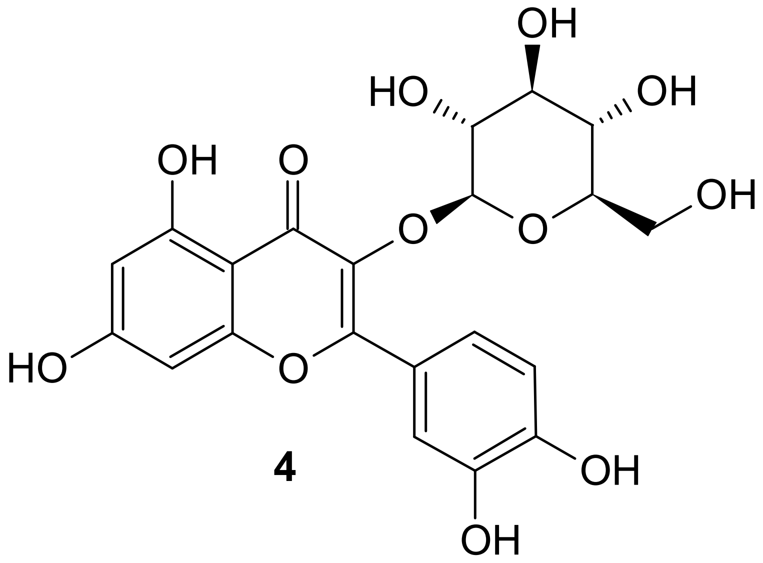

Figure S18. UV spectra of 3-*O*-β-D-(glucopyranosyl)-quercetin (**4**)

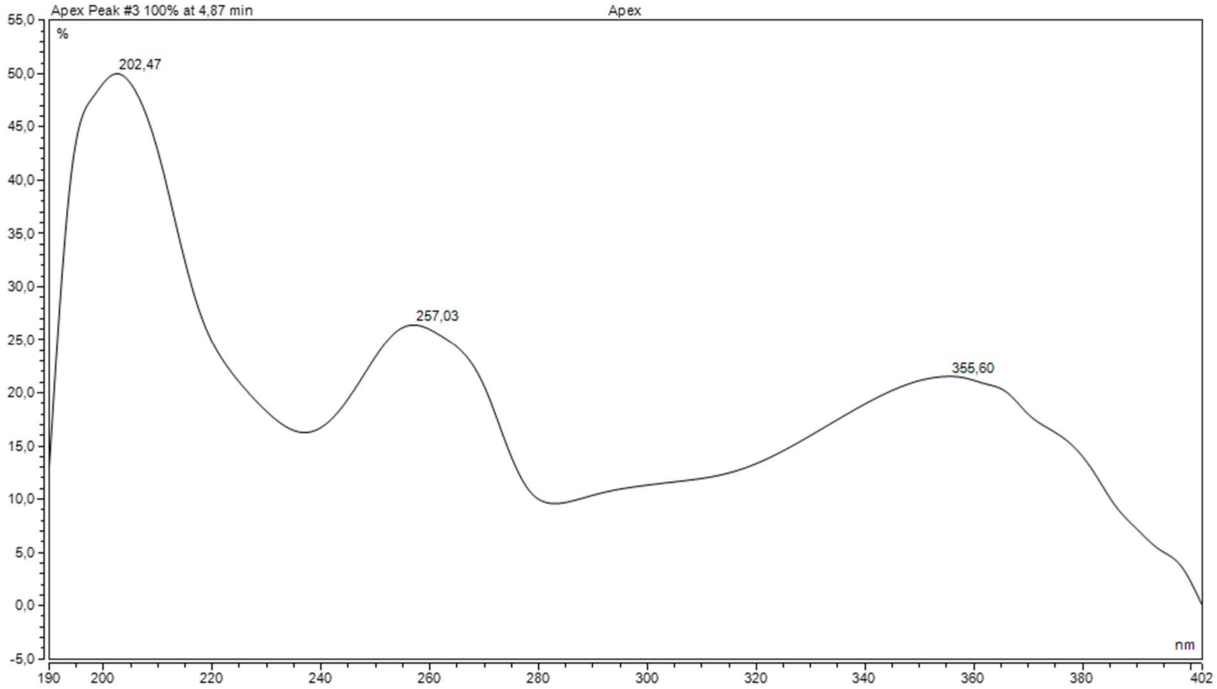

Supplementary Data 4. NMR Spectral data of 3-*O*-β-D-(glucopyranosyl)-quercetin (**4**): ^{1}H-NMR (Acetone-d_6) δH: 3.44 (1H, t, *J* = 8.8 Hz, H-4″), 3.32-3.37 (1H, m, 5′-H), 3.49-3.55 (2H, m, H-2″ and H-3″), 3.62-3.67 (1H, one of H-6″), 3.78 (1H, dd, *J* = 11.2, 2.3 Hz, one of H-6″), 5.30 (1H, d, *J* = 7.4 Hz, H-1″), 6.32 (1H, d, *J* = 2.1 Hz, H-6), 6.55 (1H, d, *J* = 2.1 Hz, H-8), 6.99 (1H, d, *J* = 8.4 Hz, H-5′), 7.62 (1H, dd, *J* = 8.4, 2.2 Hz, H-6′), 8.04 (1H, d, *J* = 2.2 Hz, H-2′). ^{13}C-NMR (DMSO-d_6) δC: 62.7 (C-6″), 75.5 (C-2″), 77.8 (C-5″), 78.1 (C-3″), 70.9 (C-4″), 94.7 (C-6), 99.7 (C-8), 105.0 (C-1″), 105.4 (C-4a), 115.8 (C-5′), 117.9 (C-2′), 122.6 (C-6′), 122.7 (C-1′), 135.6 (C-3), 145.3 (C-3′), 149.3 (C-4′), 158.0 (C-5), 158.8 (C-2), 162.9 (C-**8a**), 165.4 (C-7), 179.2 (C-4).

Figure S19. ^{1}H NMR spectra of 3-*O*-β-D-(glucopyranosyl)-quercetin (**4**) (600MHz, Acetone-*d*6)

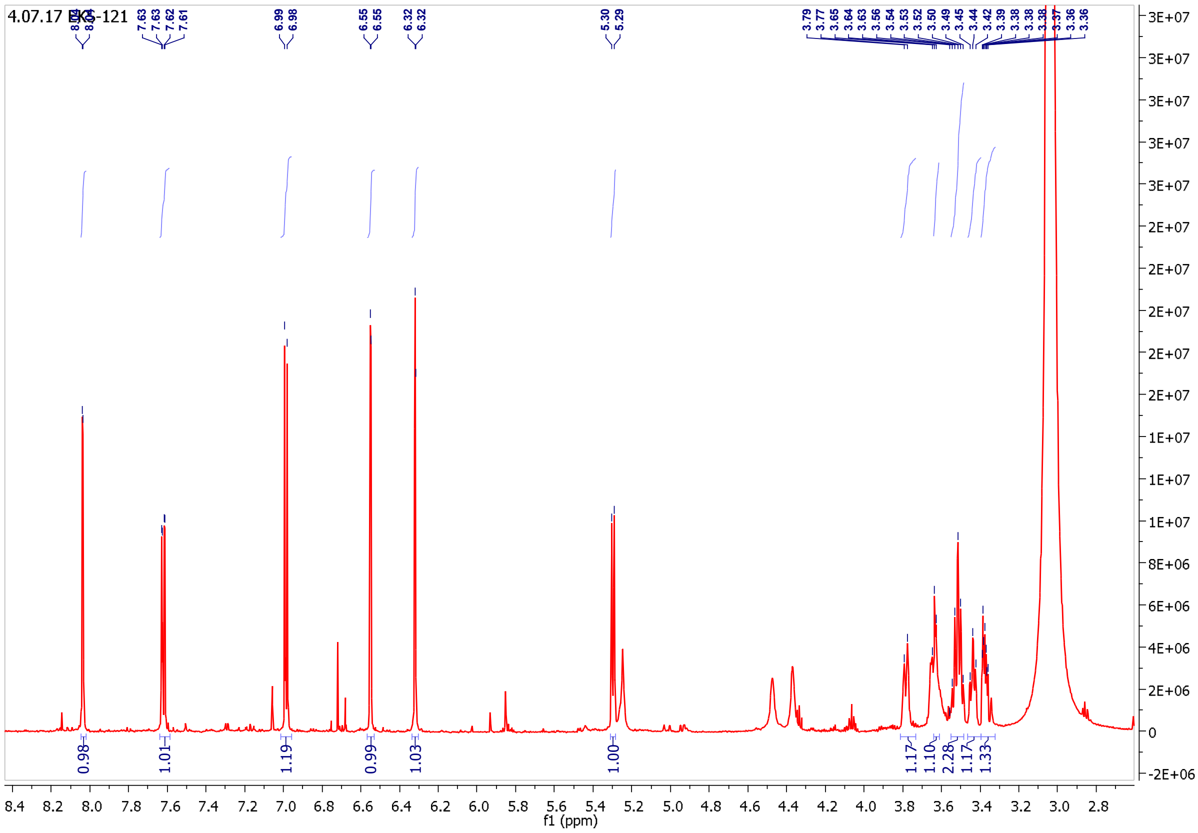

Figure S20. ^{13}C NMR spectra of 3-*O*-β-D-(glucopyranosyl)-quercetin (**4**) (151 MHz, Acetone-*d*6)

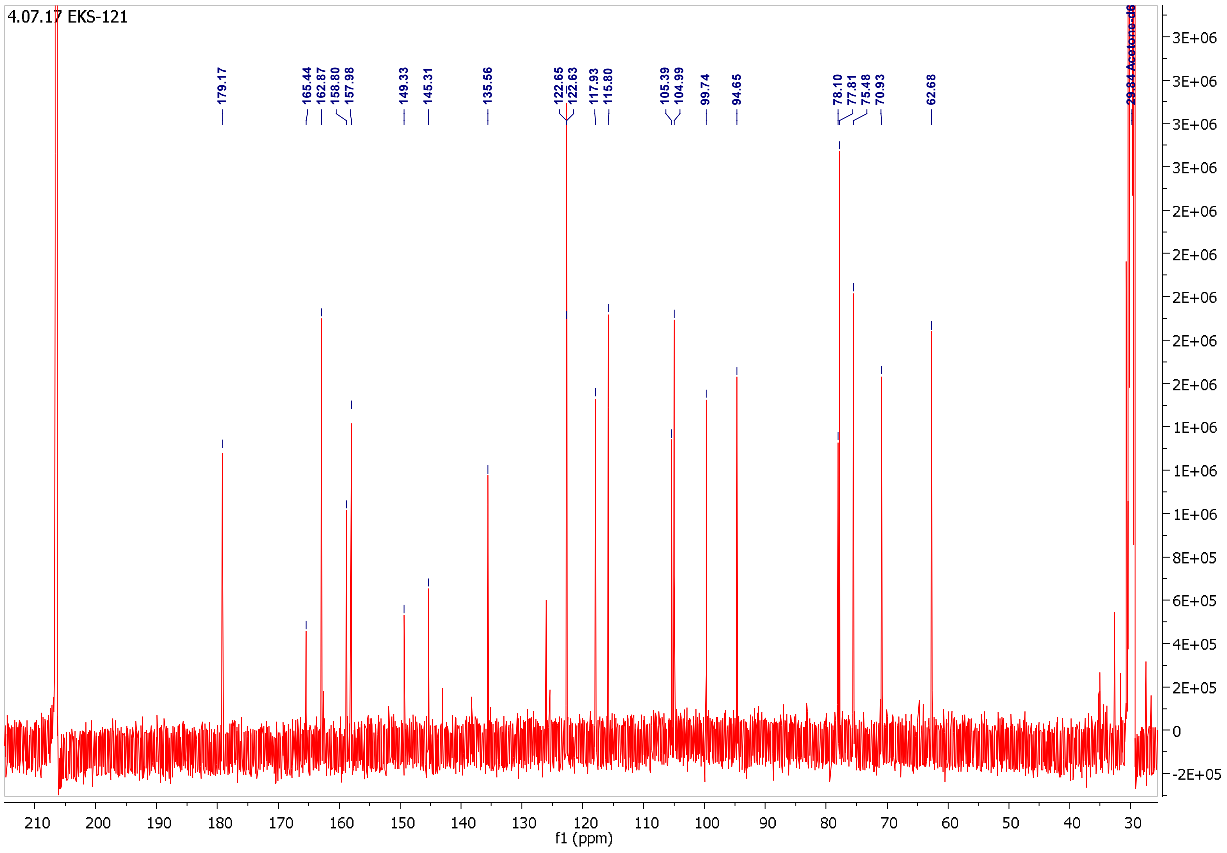

Figure S21. ^{1}H -^{1}H NMR (COSY) spectrum of quercetin 3-*O*-β-D-(glucopyranosyl)-quercetin (**4**) (600/600 MHz, Acetone-d_6)

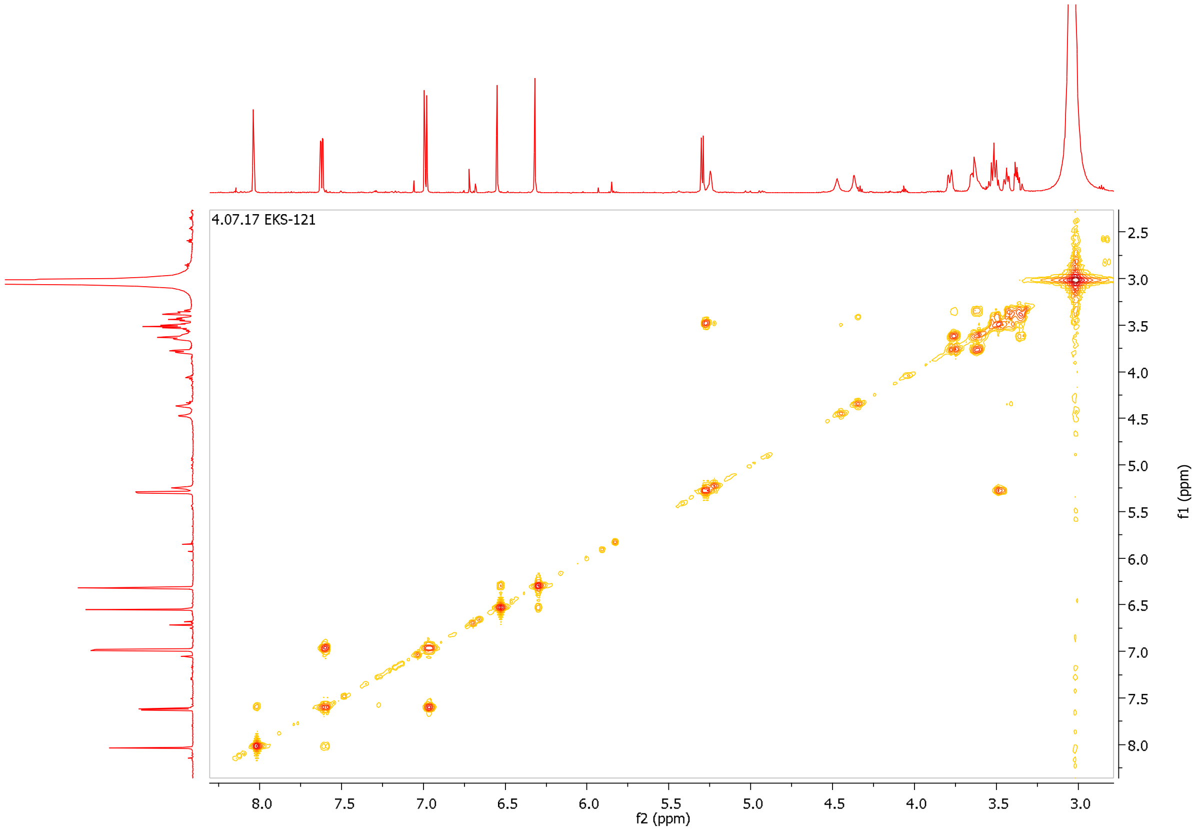

Figure S22. ^{1}H -^{13}C NMR (HSQC) spectrum of quercetin 3-*O*-β-D-(glucopyranosyl)-quercetin (**4**) (600/151 MHz, Acetone-d_6)

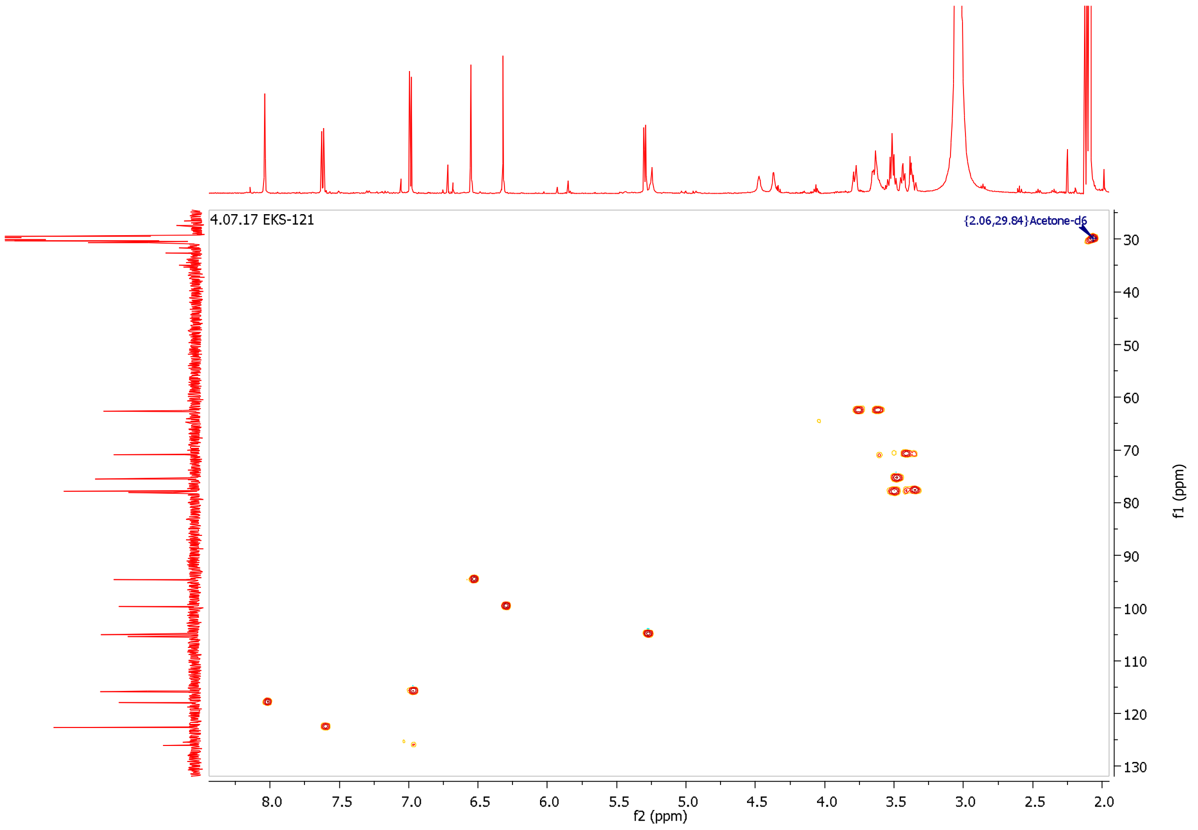

Figure S23. Selected chromatograms showing progress of quercetin (**1**) biotransformation in the cultures of entomopathogenic filamentous fungi.

Black - composition of products and unreacted substrate after the first day of biotransformation.

Blue - composition of products and unreacted substrate after the third day of biotransformation.

Pink - composition of products and unreacted substrate after the seventh day of biotransformation.

Brown - composition of products and unreacted substrate after the tenth day of biotransformation.

Retention time (t_R) of compounds **1-4**:

quercetin (**1**), t_R = 7.96 min;

7-*O*-β-D-(4″-*O*-methylglucopyranosyl)-quercetin (**2**) t_R = 4.52 min;

3-*O*-β-D-(4″-*O*-methylglucopyranosyl)-quercetin (**3**) t_R = 5.67 min;

3-*O*-β-D-(glucopyranosyl)-quercetin (**4**) t_R = 4.87 min;

a) *Beauveria bassiana* KCh J1.5

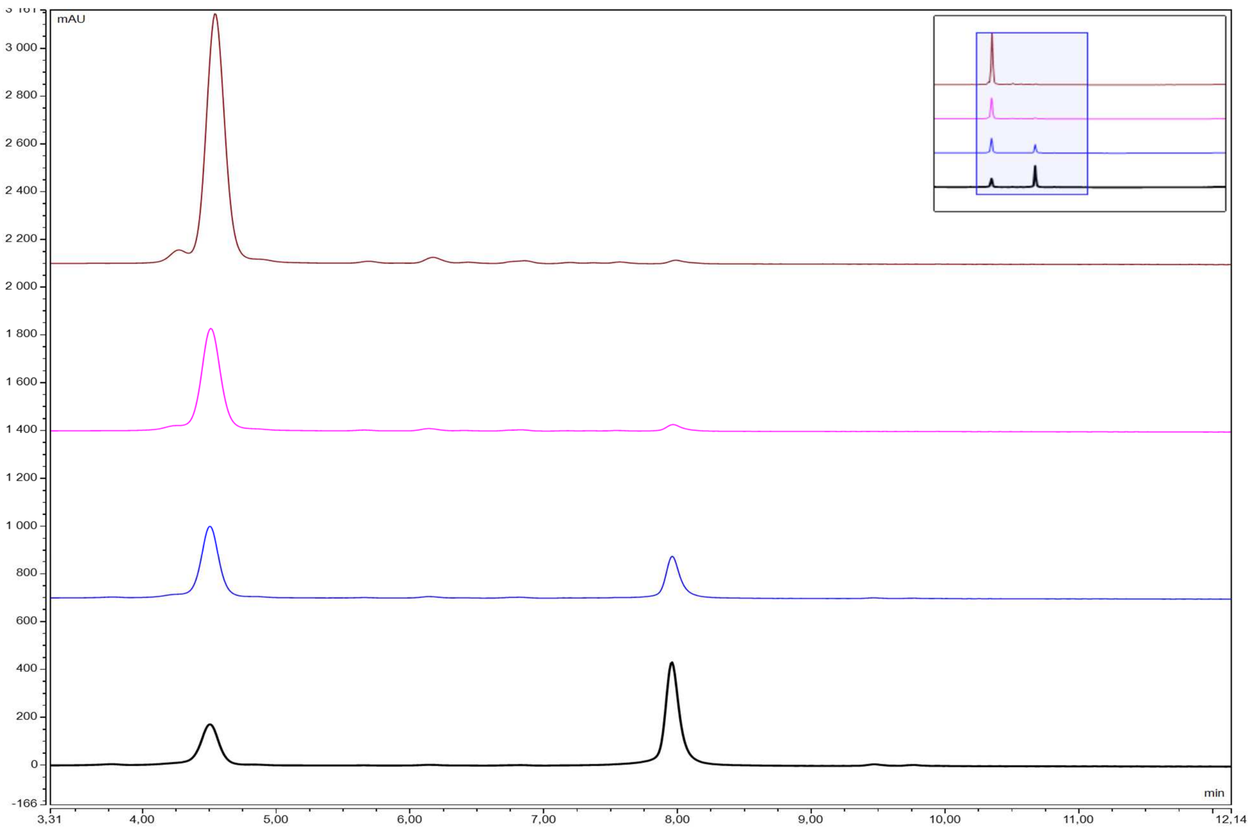

b) *Beauveria bassiana* KCh BBT

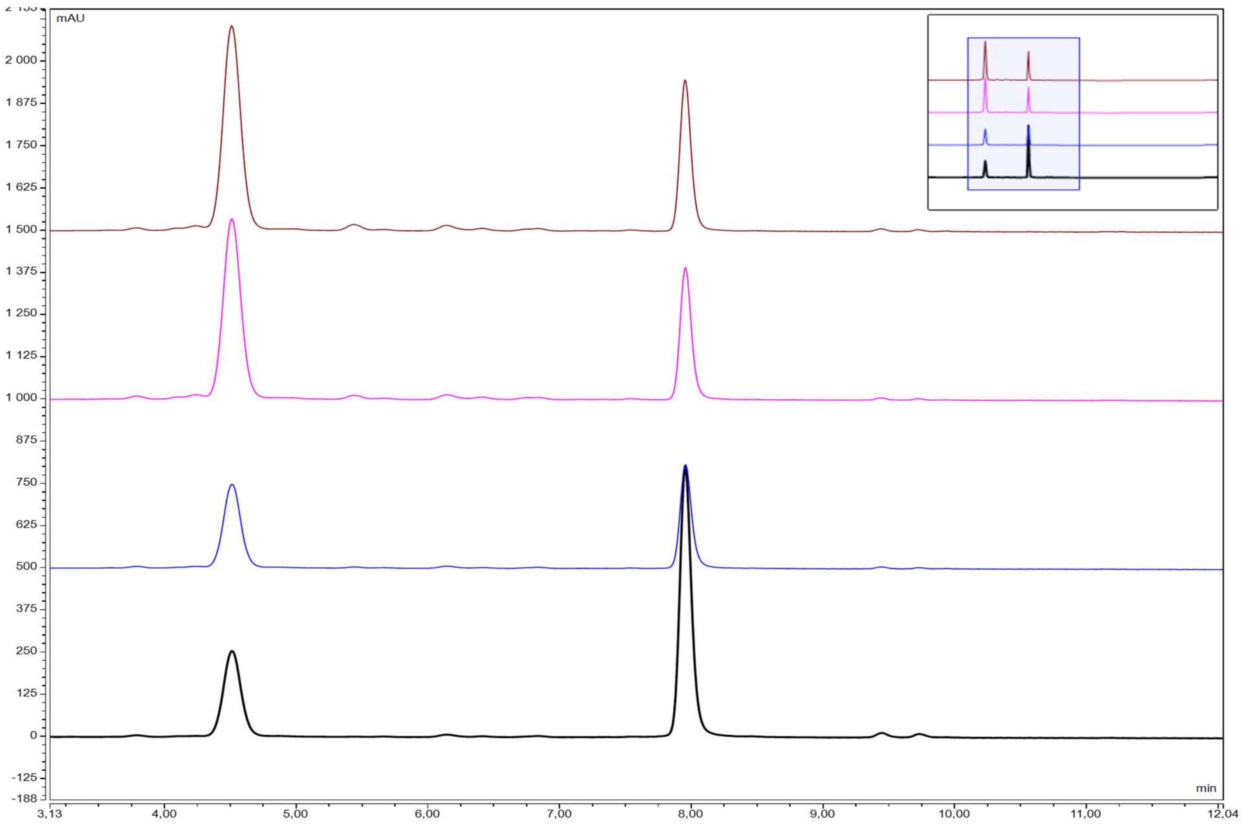

c) *Beauveria bassiana* KCh J3.2

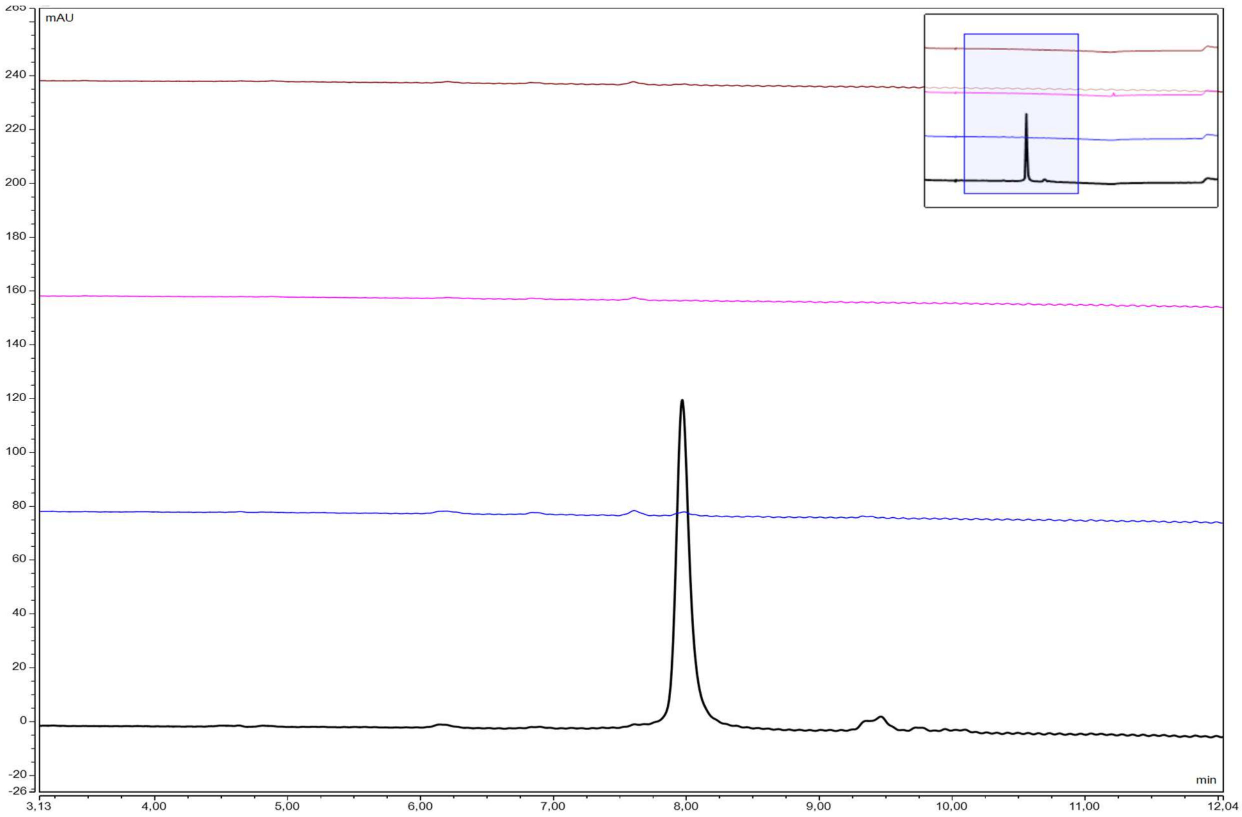

d) *Beauveria bassiana* KCh J2.1

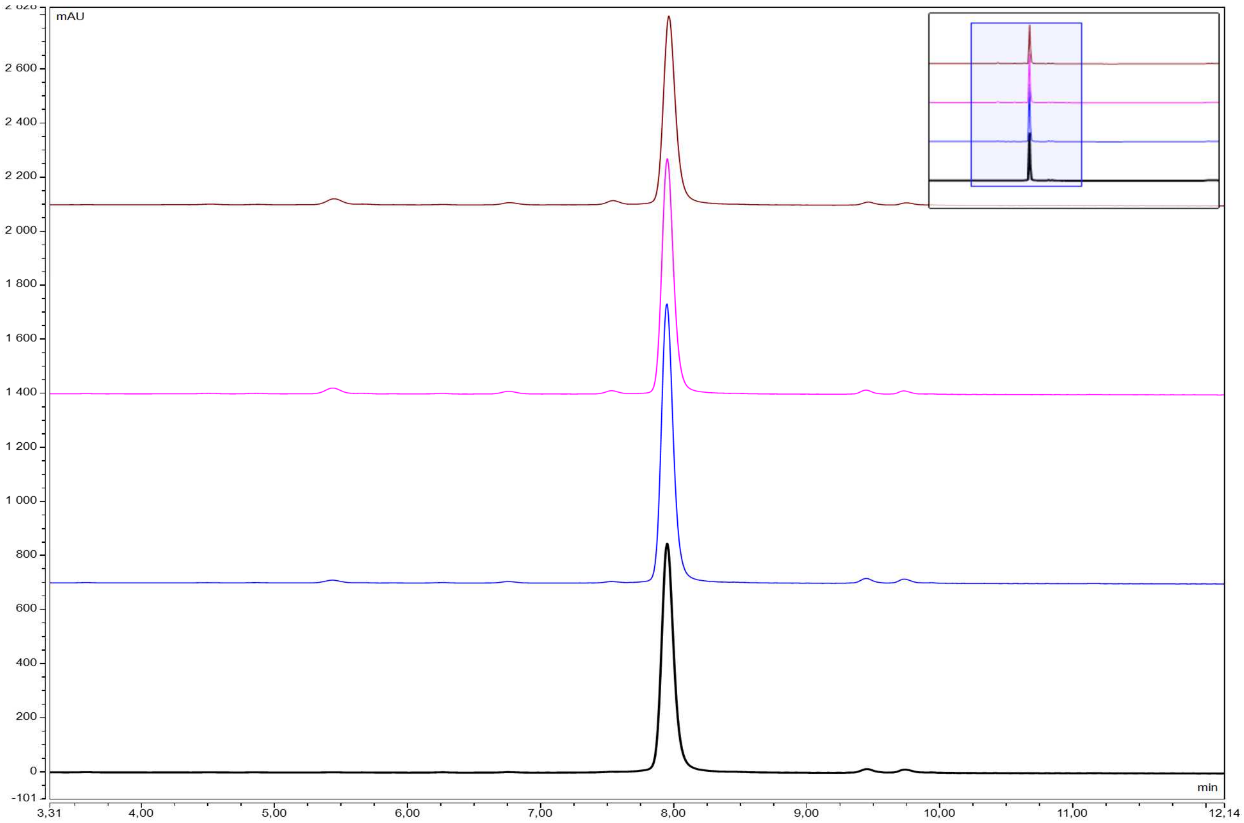

e) *Beauveria bassiana* KCh J1

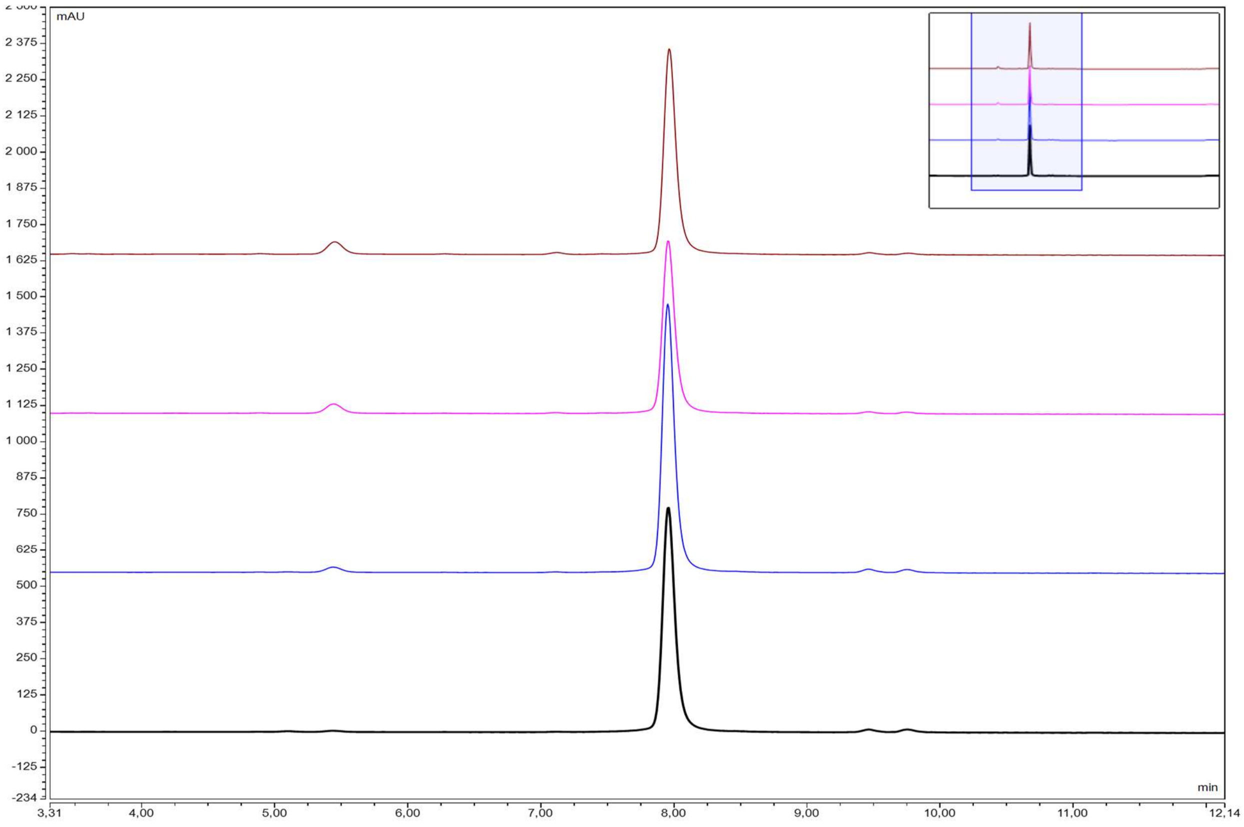

f) *Beauveria caledonica* KCh J3.3

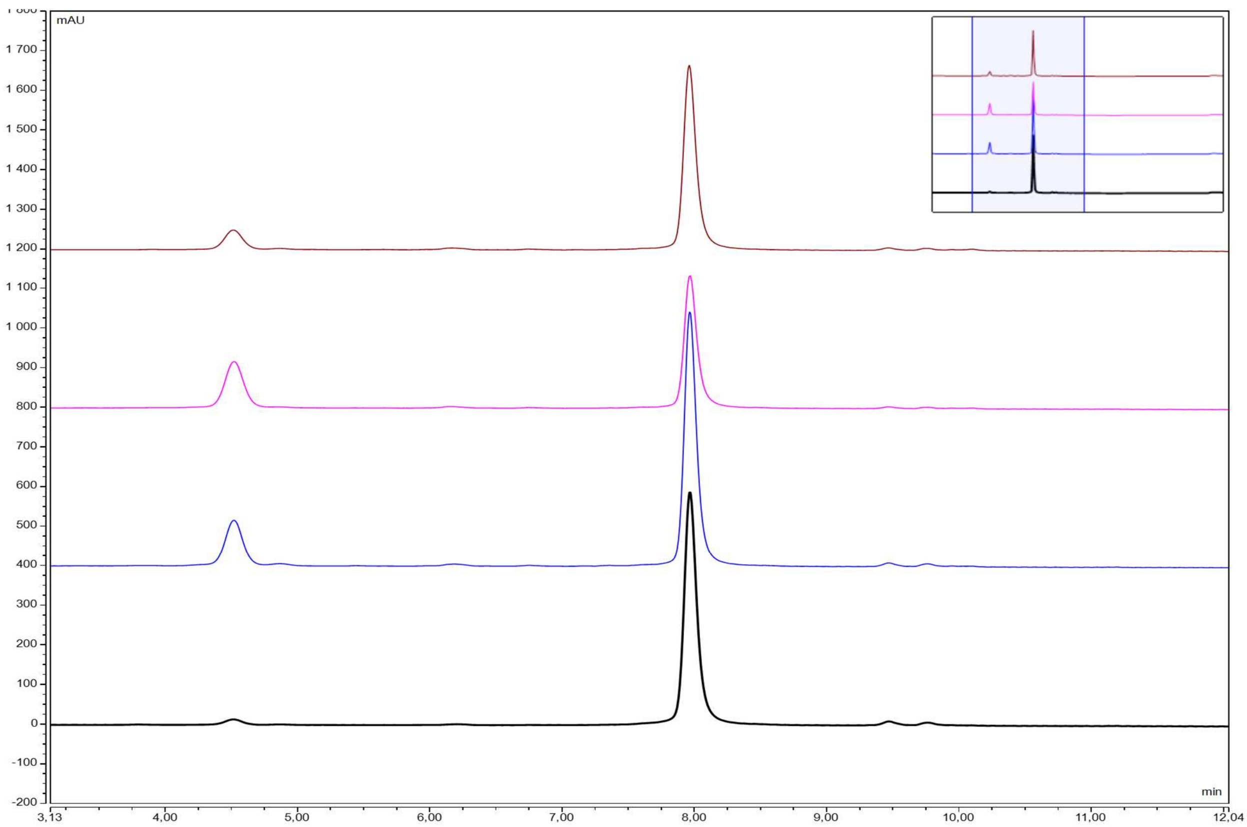

g) *Beauveria caledonica* KCh J3.4

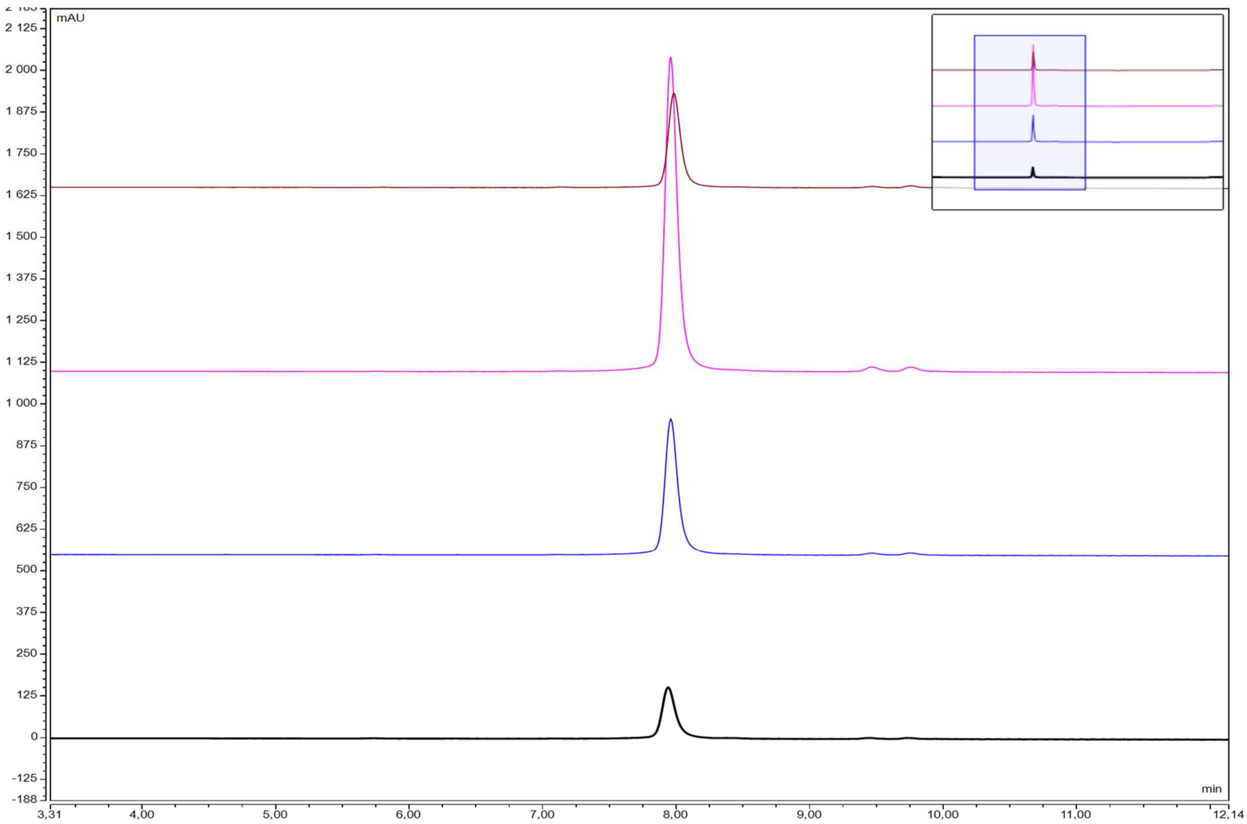

h) *Isaria farinosa* KCh KW 1.1

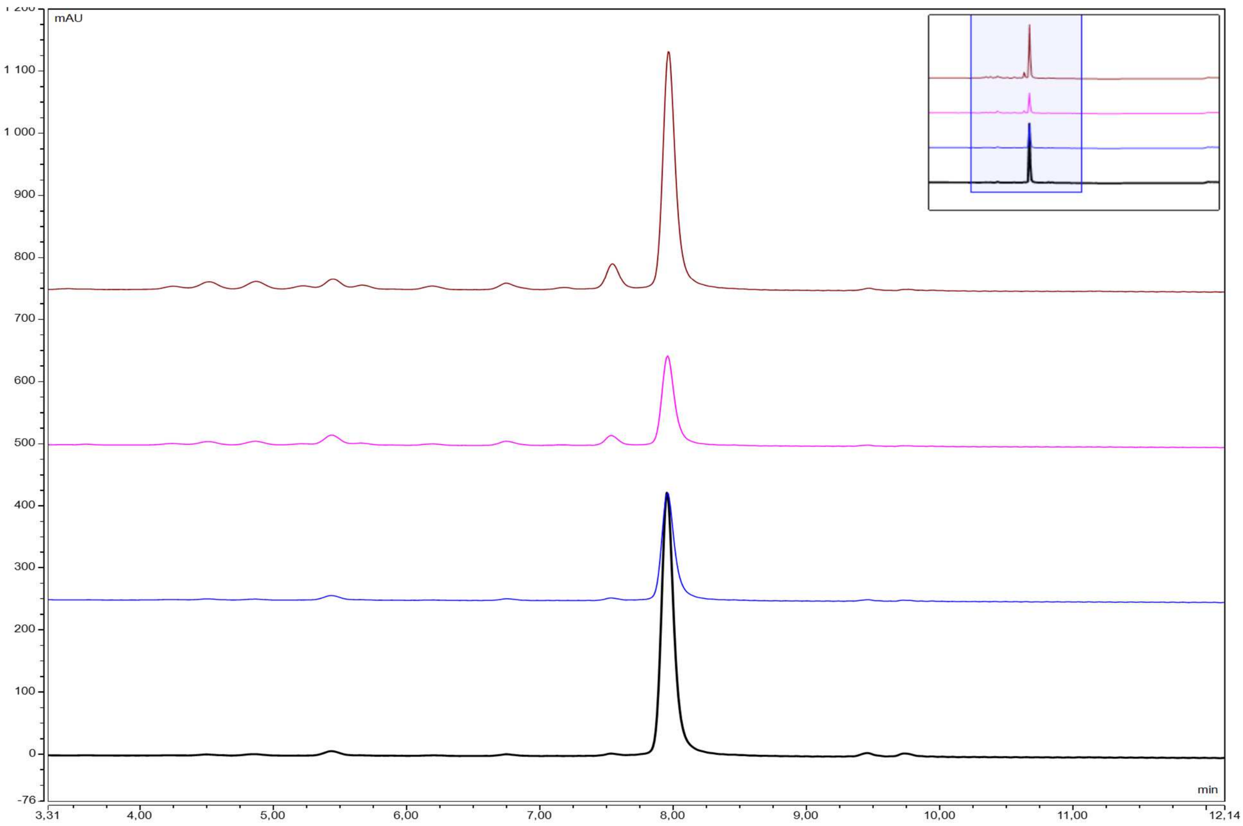

i) *Isaria tenuipes* MU35

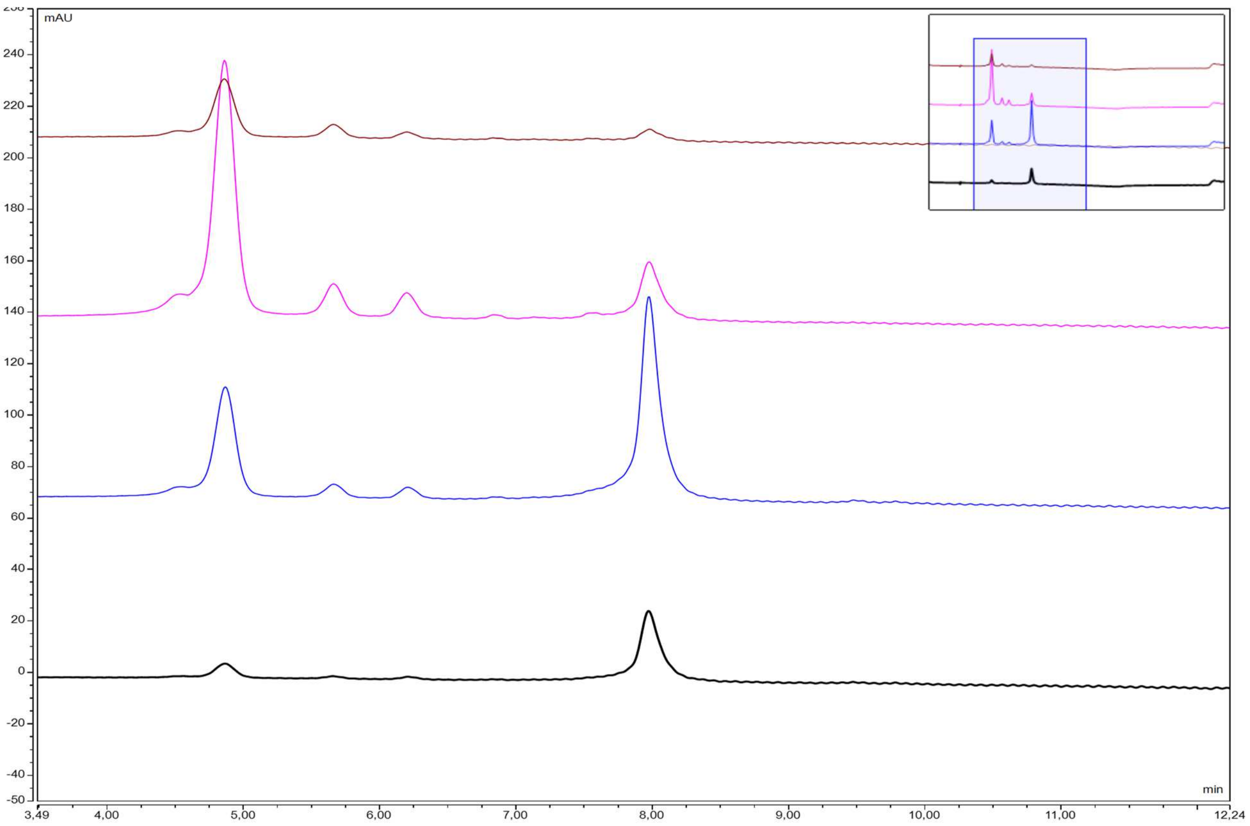

j) *Isaria tenuipes* CYS30

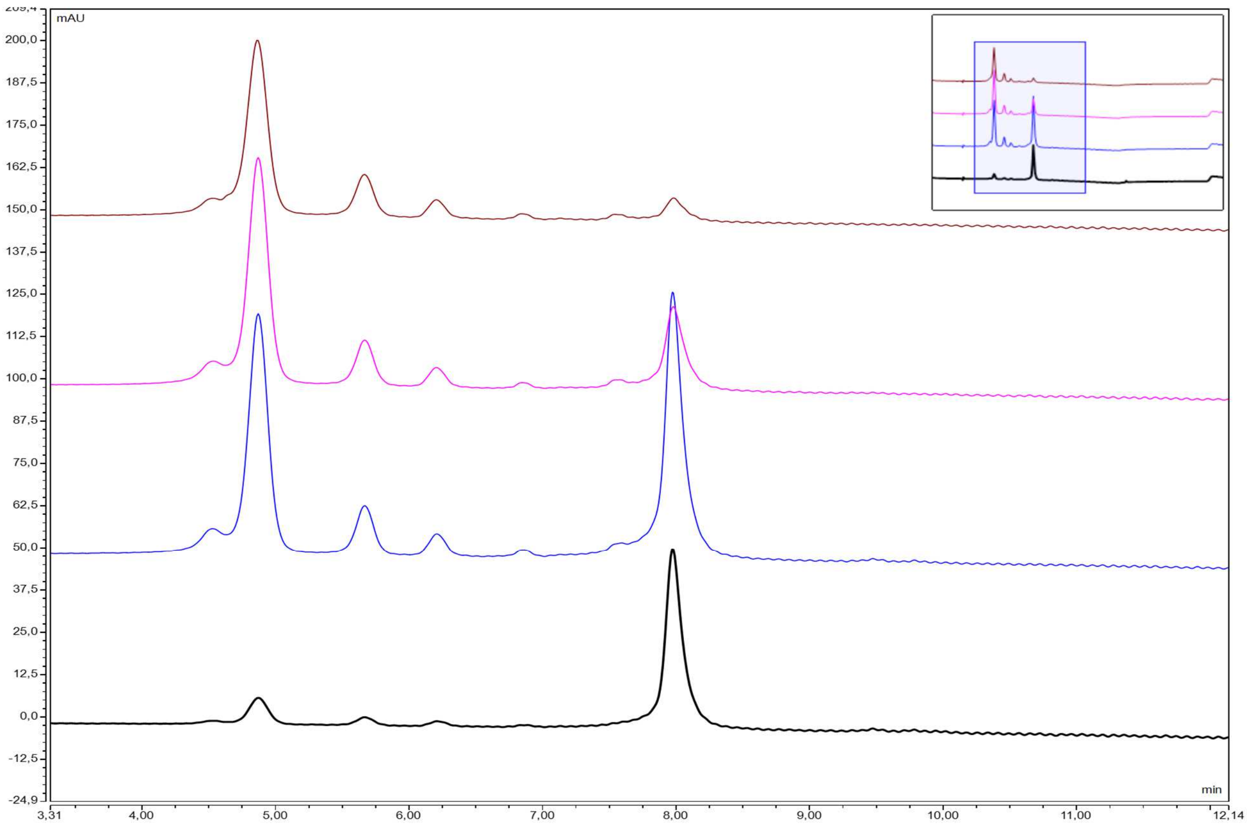

k) *Metapochonia bulbillosa* CYS17

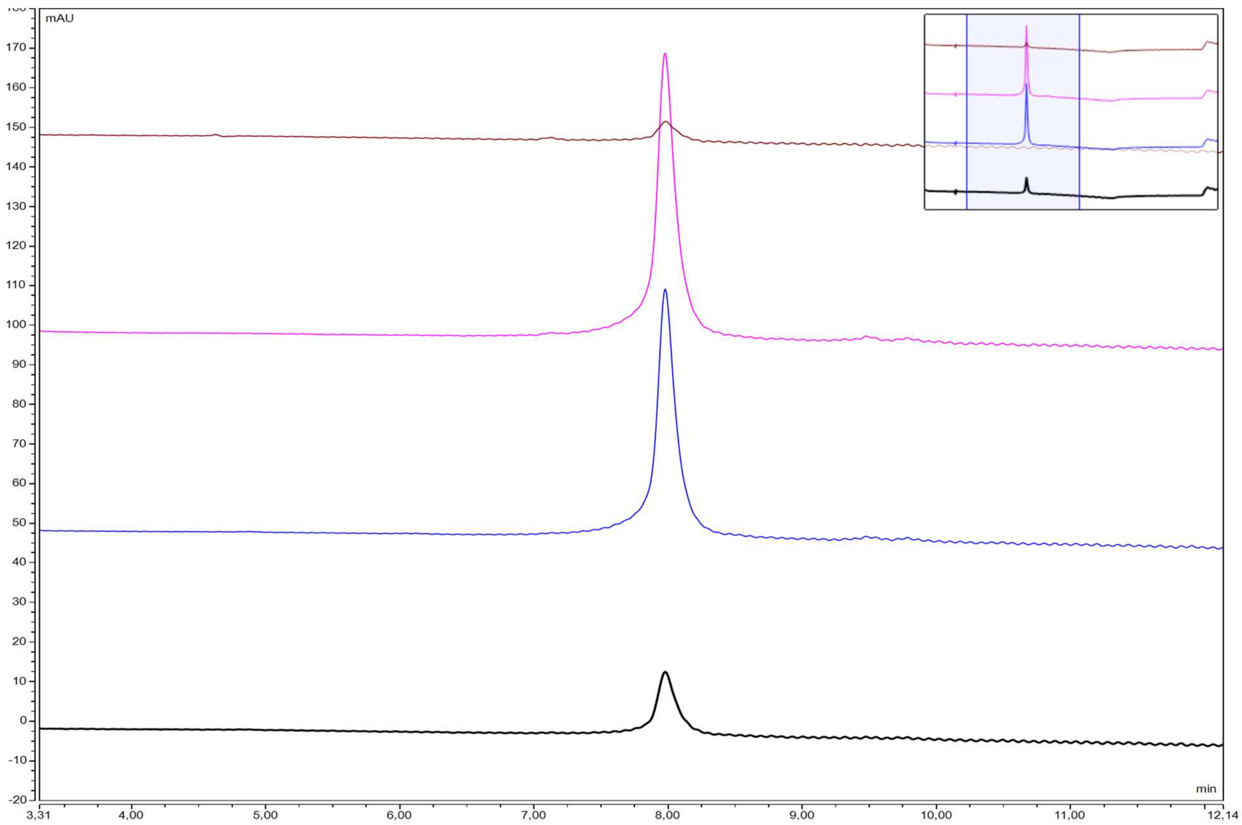

l) *Beauveria feline* ENC3

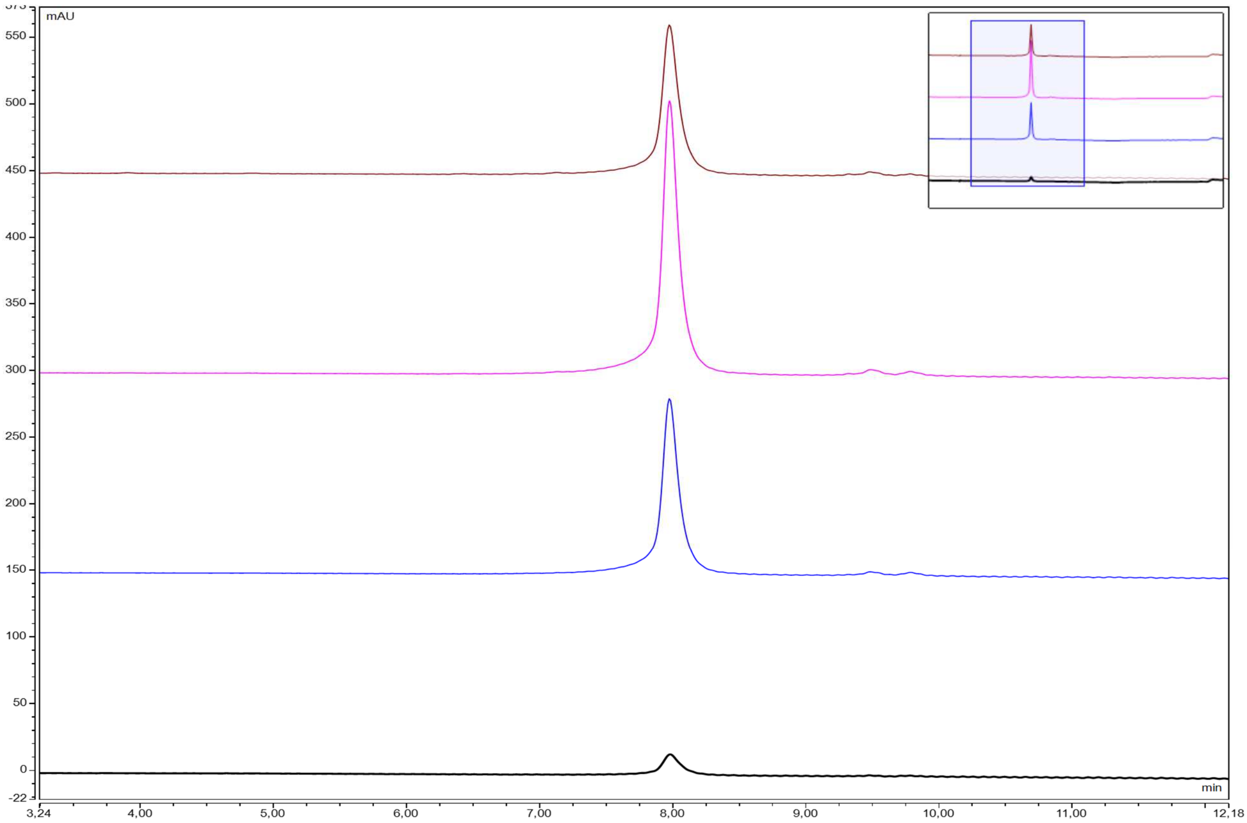

m) *Lecanicillium lecanii* DSM 63098

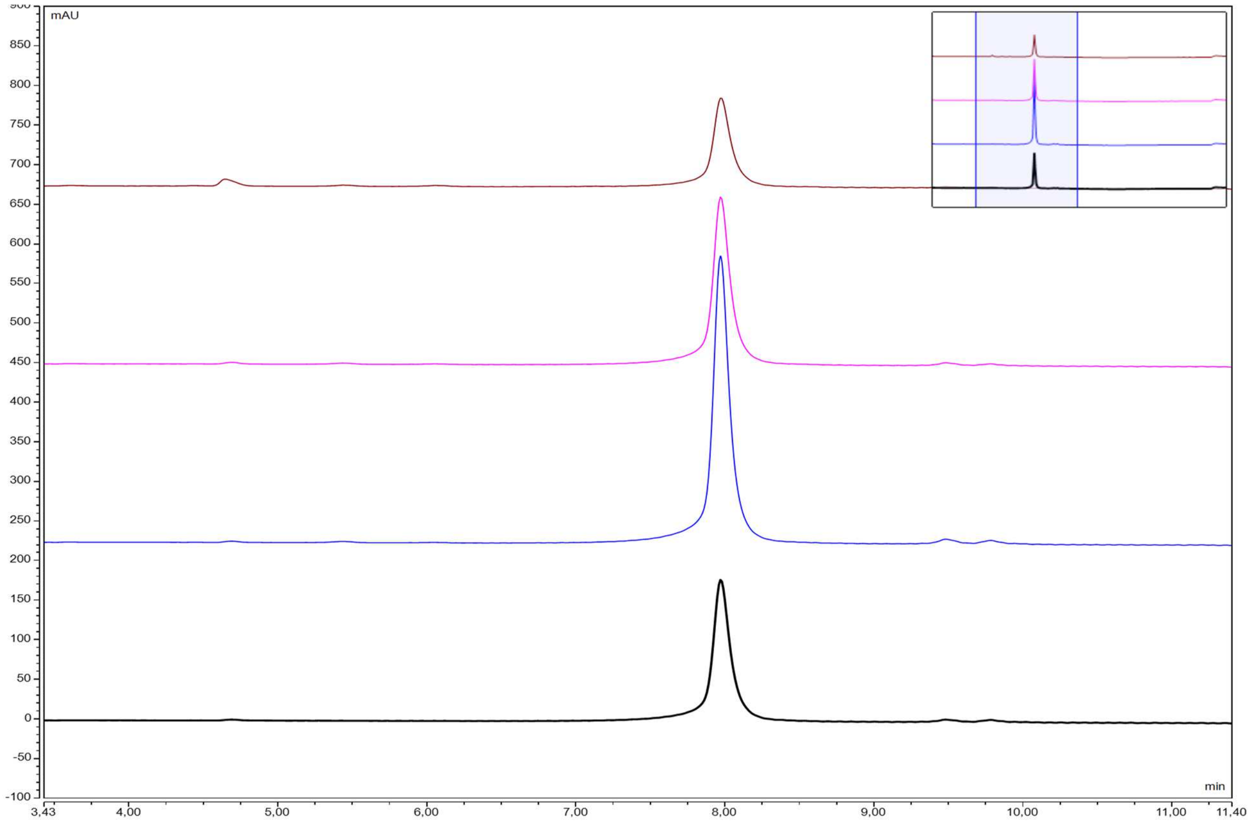

n) *Lecanicillium lecanii* NK3

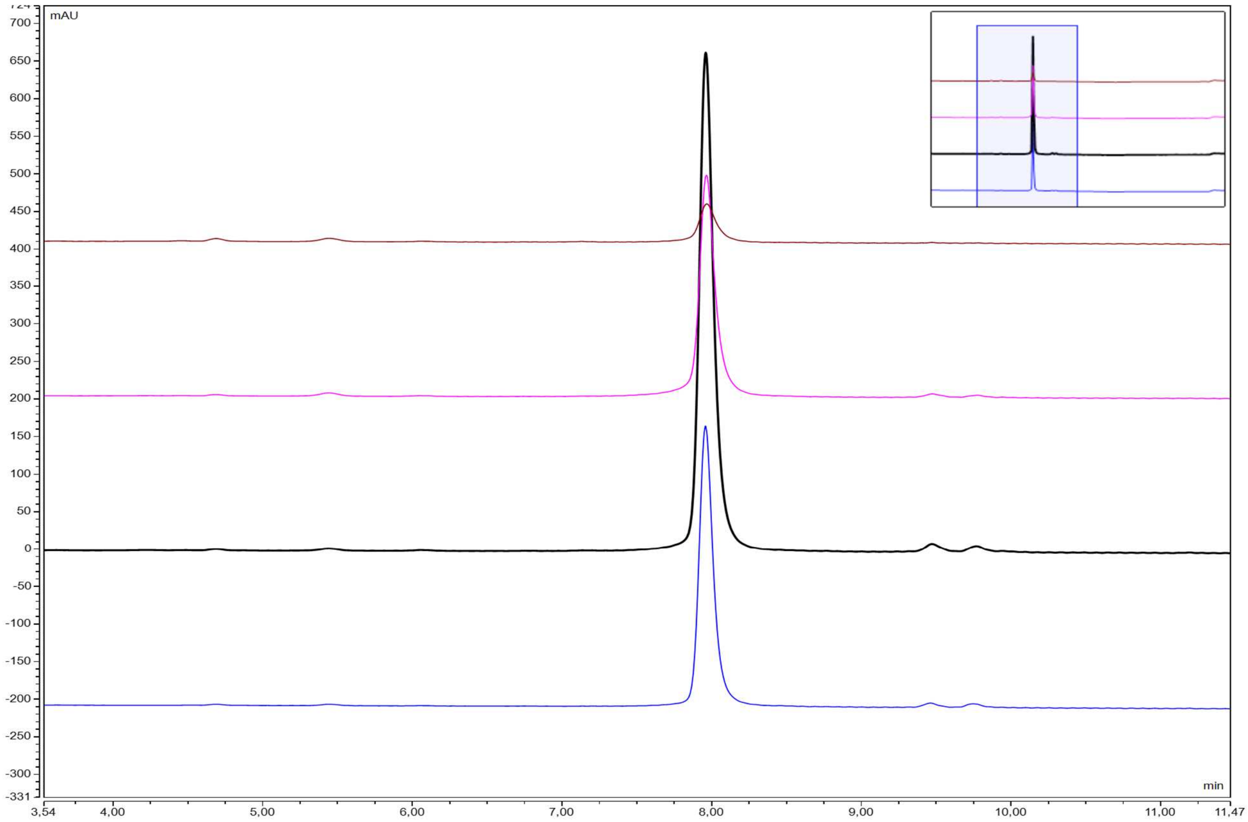

o) *Metarhizium anisopliae* MU4

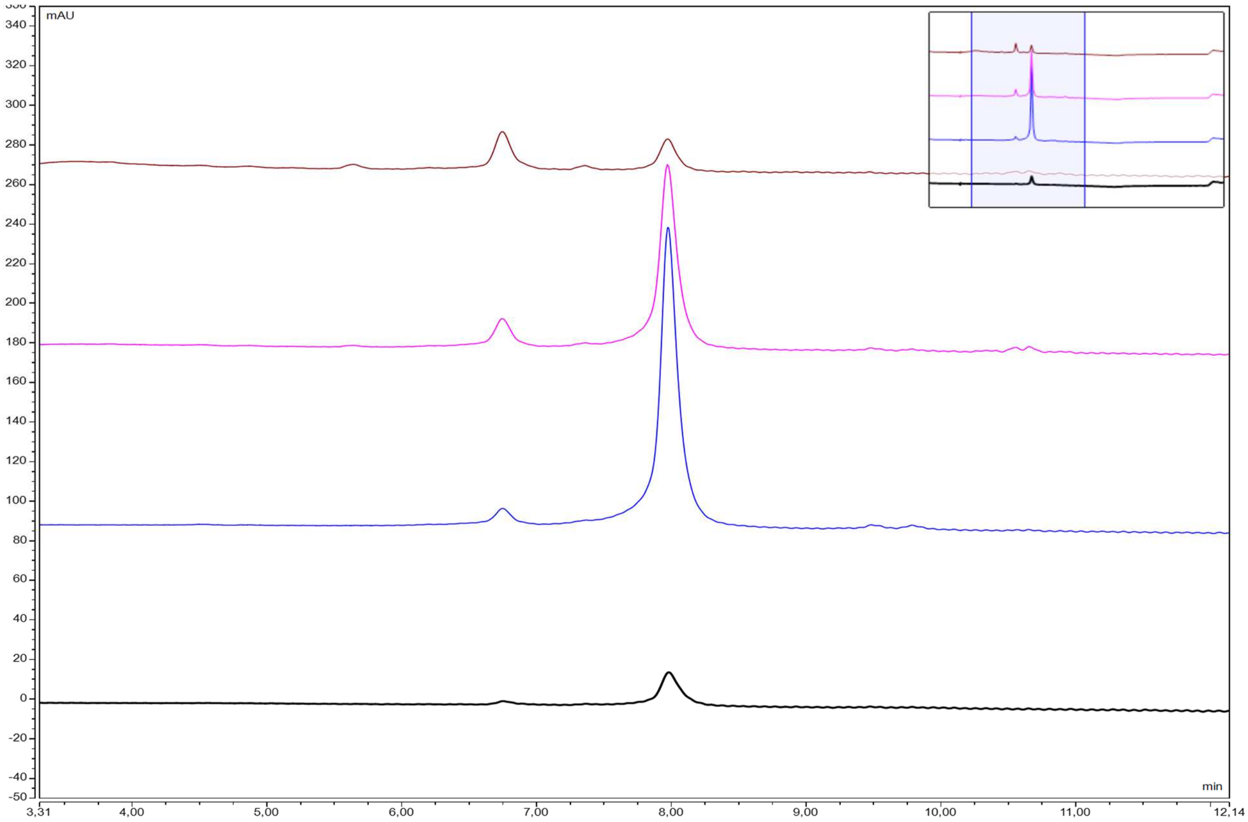

Table S2a. Microbial transformation of quercetin, HPLC conversion expressed by means and standard deviation.

Strain	Time of biotransformation [days]	Conversion [%] after 1, 3, 7 and 10 days of biotransformation									
		Substrate (1)		Products						Other products	
				2		3		4			
Beauveria bassiana KCh J1.5	1	64.3	± 3.7	35.7	± 3.7	0	-	0	-	0	-
	3	30.2	± 2.4	69.8	± 2.4	0	-	0	-	0	-
	7	3.9	± 0.3	96.1	± 0.3	0	-	0	-	0	-
	10	0.7	± 0.1	99.3	± 0.1	0	-	0	-	0	-
Beauveria bassiana KCh BBT	1	68.3	± 0.8	31.7	± 0.8	0	-	0	-	0	-
	3	44.3	± 2.5	55.7	± 2.5	0	-	0	-	0	-
	7	31.9	± 1.7	68.1	± 1.7	0	-	0	-	0	-
	10	28.0	± 2.5	72.0	± 2.5	0	-	0	-	0	-
Beauveria bassiana KCh J3.2	1	95.0	± 0.2	0	-	0	-	0	-	5.0	± 0.2
	3	0.8	± 0.8	4.4	± 0.7	0	-	0	-	94.8	± 1.5
	7	0	-	0	-	0	-	0	-	>99	-
	10	0	-	0	-	0	-	0	-	>99	-
Beauveria bassiana KCh J2.1	1	99.8	± 0.1	0.0	-	0	-	0	-	0.2	± 0.1
	3	98.3	± 0.5	0.3	± 0.2	0	-	0	-	1.4	± 0.3
	7	95.9	± 1.2	0.8	± 0.3	0	-	0	-	3.4	± 1.0
	10	91.4	± 4.0	1.9	± 1.2	0	-	0	-	6.7	± 2.9
Beauveria bassiana KCh J1	1	98.7	± 0.6	0	-	0	-	0	-	1.3	± 0.6
	3	97.7	± 0.7	0	-	0	-	0	-	2.3	± 0.7
	7	96.8	± 0.4	0	-	0	-	0	-	3.2	± 0.5
	10	95.4	± 0.6	0	-	0	-	0	-	4.6	± 0.6
Beauveria caledonica KCh J3.3	1	96.9	± 0.3	3.1	± 0.3	0	-	0	-	0	-
	3	80.3	± 2.4	19.6	± 2.4	0	-	0	-	0	-
	7	67.6	± 3.3	32.4	± 3.3	0	-	0	-	0	-
	10	49.7	± 4.0	13.0	± 0.5	0	-	0	-	37.2	± 4.1
Beauveria caledonica KCh J3.4	1	99.8	± 0.1	0	-	0	-	0	-	0.2	± 0.1
	3	99.6	± 0.0	0	-	0	-	0	-	0.4	± 0.1
	7	99.3	± 0.2	0	-	0	-	0	-	0.7	± 0.2
	10	99.0	± 0.2	0	-	0	-	0	-	1.0	± 0.2
Isaria farinosa KCh KW 1.1.	1	95.8	± 0.9	0.4	± 0.1	0.1	± 0.0	0.6	± 0.1	3.1	± 0.8
	3	92.0	± 1.5	1.5	± 0.4	0.8	± 0.3	1.9	± 0.3	3.8	± 0.7
	7	86.2	± 0.1	2.9	± 0.3	1.5	± 0.3	3.4	± 0.0	6.0	± 0.3
	10	79.6	± 0.8	2.8	± 0.2	0.8	± 0.1	3.5	± 0.2	13.4	± 0.9
Isaria tenuipes MU35	1	79.8	± 1.6	1.7	± 0.0	2.0	± 0.3	16.4	± 1.4	0	-
	3	61.0	± 2.8	2.6	± 0.2	3.3	± 0.2	33.1	± 2.4	0	-
	7	18.6	± 5.1	4.2	± 1.2	7.0	± 1.1	70.2	± 3.0	0	-
	10	10.4	± 0.3	5.9	± 0.9	12.1	± 2.2	67.2	± 0.8	4.4	± 1.8
Isaria tenuipes CYS30	1	82.2	± 1.6	1.5	± 0.3	3.2	± 0.7	13.2	± 0.8	0	-
	3	43.3	± 2.6	4.2	± 0.8	6.8	± 1.9	45.6	± 3.4	0	-
	7	23.5	± 6.1	5.9	± 0.2	9.8	± 2.1	60.8	± 4.0	0	-
	10	11.0	± 2.5	7.0	± 0.8	13.1	± 3.1	69.0	± 1.7	0	-

Table S2b. Microbial transformation of quercetin, HPLC conversion expressed by means and standard deviation (continuation).

Strain	Time of biotransformation [days]	Conversion [%] after 1, 3, 7 and 10 days of biotransformation				
		Substrate (1)	Products			Other products
			2	3	4	
Metapochonia bulbillosa CYP17	1	98.9 ± 0.5	0 -	0 -	0 -	1.1 ± 0.5
	3	98.5 ± 0.4	0 -	0 -	0 -	1.8 ± 0.7
	7	97.0 ± 0.2	0 -	0 -	0 -	3.0 ± 0.2
	10	96.0 ± 0.6	0 -	0 -	0 -	4.0 ± 0.6
Beauveria felina ENC3	1	>99 -	0 -	0 -	0 -	<1 -
	3	>99 -	0 -	0 -	0 -	<1 -
	7	>99 -	0 -	0 -	0 -	<1 -
	10	>99 -	0 -	0 -	0 -	<1 -
Lecanicillium lecanii DSM 63098	1	>99 -	0 -	0 -	0 -	<1 -
	3	>99 -	0 -	0 -	0 -	<1 -
	7	>99 -	0 -	0 -	0 -	<1 -
	10	>99 -	0 -	0 -	0 -	<1 -
Lecanicillium lecanii NK3	1	>99 -	0 -	0 -	0 -	<1 -
	3	>99 -	0 -	0 -	0 -	<1 -
	7	>99 -	0 -	0 -	0 -	<1 -
	10	>99 -	0 -	0 -	0 -	<1 -
Metarhizium anisopliae MU4	1	96.6 ± 1.4	0 -	0 -	0 -	3.4 ± 1.4
	3	86.8 ± 6.3	0 -	0 -	0.1 ± 0.1	15.4 ± 6.8
	7	48.9 ± 8.2	0 -	0 -	0.6 ± 0.2	50.5 ± 8.2
	10	10.1 ± 2.4	0 -	0 -	4.2 ± 0.8	85.7 ± 2.5

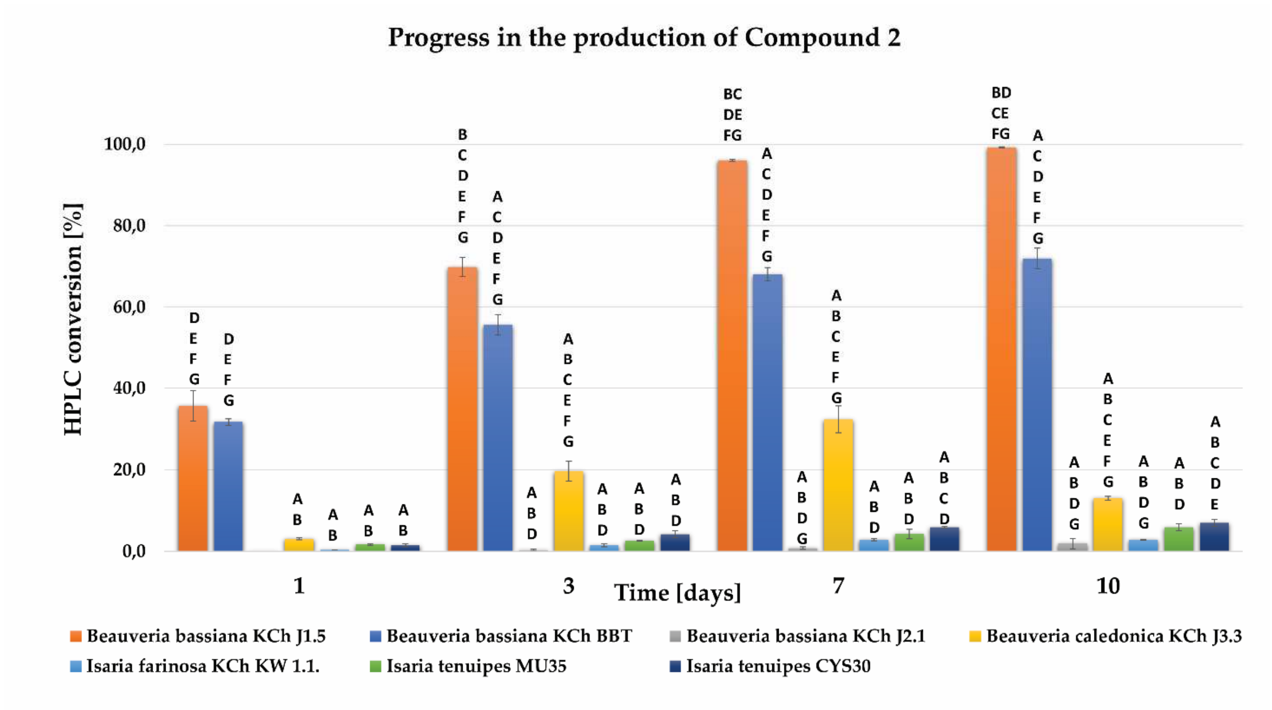

Figure S24. Progress in production of compound **2** by entomopathogenic fungi.

Statistically significant at p value < 0.05: **A** – vs obtaining of compound **2** by *Beauveria bassiana* KCh J1.5; **B** – vs obtaining of compound **2** by *Beauveria bassiana* KCh BBT; **C** – vs obtaining of compound **2** by *Beauveria bassiana* KCh J2.1; **D** – vs obtaining of compound **2** by *Beauveria caledonica* KCh J3.3; **E** – vs obtaining of compound **2** by *Isaria farinosa* KCh KW 1.1; **F** – vs obtaining of compound **2** by *Isaria tenuipes* MU35; **G** – vs obtaining of compound **2** by *Isaria tenuipes* CYS30

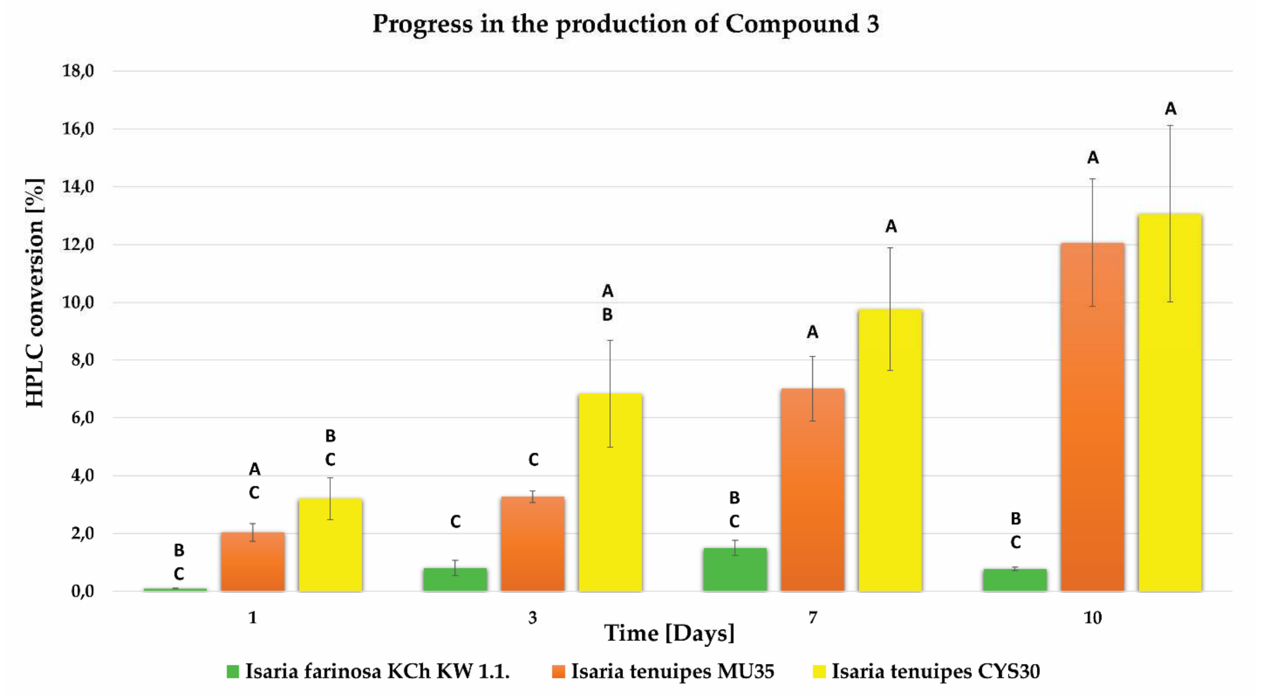

Figure S25. Progress in production of compound **3** by entomopathogenic fungi.

Statistically significant at p value < 0.05: **A** – vs obtaining of compound **3** by *Isaria farinosa* KCh KW 1.1; **B** – vs obtaining of compound **3** by *Isaria tenuipes* MU35; **C** – vs obtaining of compound **3** by *Isaria tenuipes* CYS30

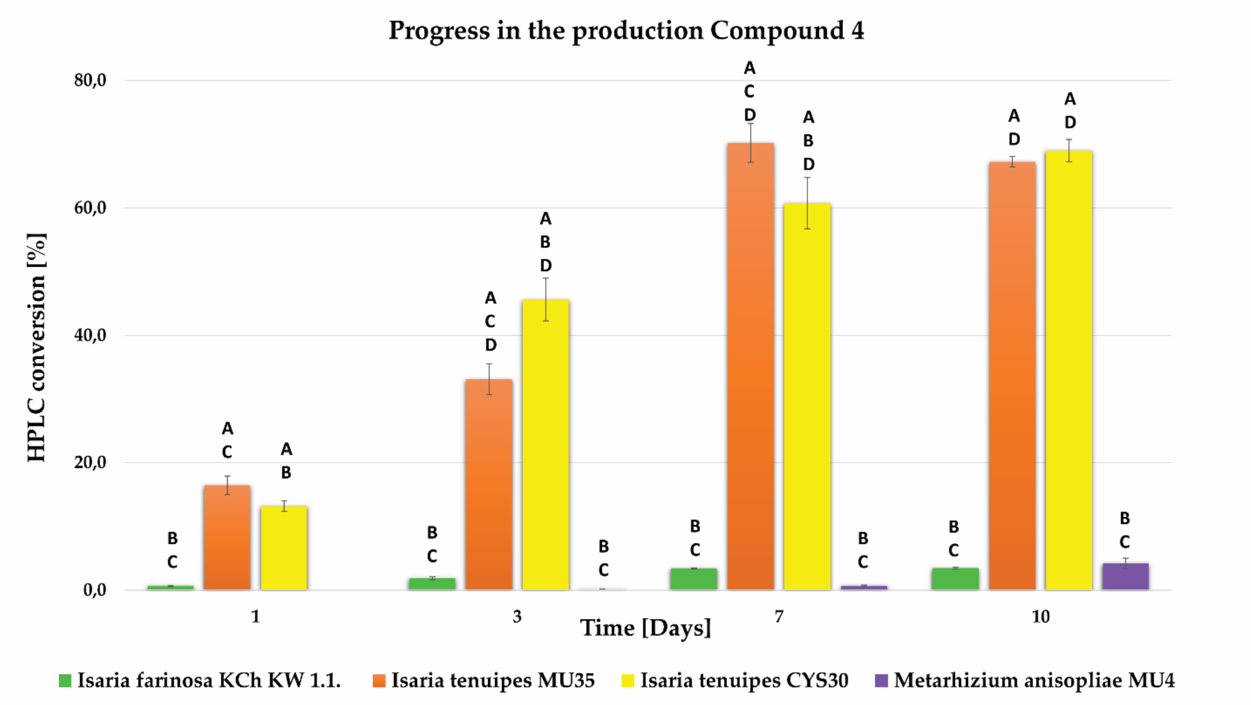

Figure S26. Progress in production of compound **4** by entomopathogenic fungi.

Statistically significant at *p* value < 0.05: **A** – vs obtaining of compound **4** by *Isaria farinosa* KCh KW 1.1; **B** – vs obtaining of compound **4** by *Isaria tenuipes* MU35; **C** – vs obtaining of compound **4** by *Isaria tenuipes* CYS30; **D** – vs obtaining of compound **4** by *Metarhizium anisopliae* MU4
